# Short-term impact of low air pressure on plants’ functional traits

**DOI:** 10.1371/journal.pone.0317590

**Published:** 2025-01-15

**Authors:** Silvia Lembo, Georg Niedrist, Bouchra El Omari, Paul Illmer, Nadine Praeg, Andreas Meul, Matteo Dainese

**Affiliations:** 1 Institute for Alpine Environment, Eurac Research, Bolzano, Bozen, Italy; 2 Department of Microbiology, Universität Innsbruck, Innsbruck, Austria; 3 Department of Biotechnology, University of Verona, Verona, Italy; University of Udine: Universita degli Studi di Udine, ITALY

## Abstract

Lower atmospheric pressure affects biologically relevant physical parameters such as gas partial pressure and concentration, leading to increased water vapor diffusivity and greater soil water content loss through evapotranspiration. This might impact plant photosynthetic activity, resource allocation, water relations, and growth. However, the direct impact of low air pressure on plant physiology is largely unknown. This study examined the effects of low air pressure, alone and combined with two water inputs, on different functional traits of three plant species transplanted from montane grasslands at 1,500 m a.s.l. during the first four weeks of their early phenological stage: *Trifolium pratense*, *Hieracium pilosella*, and B*rachypodium rupestre*. Using the terraXcube Ecotron facility which can simulate different climatic conditions, we isolated the effect of air pressure from those of other, related environmental factors (temperature, humidity, and solar radiation) by simulating three different elevations with corresponding air pressures: 1,500 m a.s.l. (85 kPa, *control scenario*), 2,500 m a.s.l. (75 kPa), and 4,000 m a.s.l. (62 kPa) and we used two different water regimes to observe the combined effect of low air pressure and the impact of varying water inputs on plants. In *T*. *pratense* and *H*. *pilosella*, we observed an increase in stomatal conductance but a reduction in aboveground biomass at the lowest pressure compared to the control scenario after four weeks of incubation. Contrastingly, *B*. *rupestre* showed an interactive effect of air pressure and water treatment on chlorophyll and biomass nitrogen content, which were reduced under higher soil water conditions at 85kPa. This study serves as an initial step in isolating the specific impact of air pressure on plant physiology, demonstrating the potential of the facility for future research. The mixed response patterns across species highlight that atmospheric pressure could be a driving factor to consider when assessing plant responses along elevational gradient.

## 1 | Introduction

Climate change affects the composition and function of ecosystems by influencing the velocity of phenological and community distributional shifts, interspecific differences, recolonisation processes, and the abundance of many species [1]. It is evident that increasing temperatures drive upward shifts in the elevation range [2] of many plant species from different mountain regions, including the Alps [3–6]. In this context, elevation-related temperature reduction is often considered the main driving factor influencing, directly and/or indirectly, the distribution and physiology of different organisms in mountain ecosystems [7]. However, other physical parameters such as UV radiation or snow cover duration co-vary with temperature and reduced air pressure is often neglected [7].

With ongoing climate change, the warming rate increases with elevation [8] and the joint variation of temperature and air pressure could be altered along elevational gradients; however, the consequences of this decoupling for plants and ecosystems are unknown. While temperature is consistently projected to increase, air pressure will remain almost constant [9]. This means that organisms that migrate upward to track their pre-warming temperatures may encounter a decrease in air pressure as they move to increasingly higher elevations [10]. Consequently, reduced air pressure poses a potentially novel environmental challenge for upward-migrating organisms if they persist at high elevations as climate change continues [11].

With a decrease in air pressure, the partial pressure of all atmospheric gases (CO_2_, O_2_, and water vapor) diminish [12]. This decline in the availability of gases is theorized to have important consequences on living organisms, the extent of which is not well known [13], although it can significantly influence respiratory activity in animals, leaf gas exchange in plants, and metabolic activities of certain microorganisms [13–15]. Specifically, plants can react differently to novel atmospheric conditions due to their different physiological tolerances and adaptability to, for example, decreased CO_2_ and O_2_ partial pressures [16, 17]. Their responses to novel conditions are shaped by their morphological and eco-physiological traits [18]. These adaptations can influence plant performance and fitness by modifying critical physiological processes, such as water use efficiency, resource allocation, and growth. In some cases, they may even alter the photosynthetic pathway. e.g. reduced air pressure (30 kPa) increased leaf chlorophyll content and biomass production in lettuce plants compared with plants living at sea level pressure [19]. Similarly, wheat leaves might enhance stomatal conductance leading to an increase in CO_2_ assimilation, and carbohydrate metabolism [20, 21]. Moreover, Midolo et al. 2019 [22] found that specific leaf area (SLA) diminished with decreasing air pressure, a greater reduction for herbaceous species than for woody species.

However, the diminished air pressure might also enhance the diffusivity of gases in the air, counteracting the lower absolute concentration of atmospheric gases [12]. Indeed, while the decrease of water vapor in the air enhances the gradient of water vapor between leaves and the atmosphere, the rised diffusivity of water vapor leads to an increase in the transpiration rate [12]. The extent of this higher transpiration on plant water status depends on environmental conditions like the air-to-leaf temperature gradient [23], but also on the inherent characteristics of plant species such as tolerance to stress, plant life strategy, etc [24]. In addition, soil evaporation is also enhanced in reduced air pressure conditions. A lower soil water content, through higher evaporation, will drop the aboveground biomass [25] and primary plants´ productivity [26]. Thus, it is important to understand the effects of lower air pressure on plants combined with different water regimes.

In this context, only a limited number of studies have attempted to understand the direct effects of lower atmospheric pressure alone [16, 27, 28]. This is because of the challenging technical requirements for truly separating highly intercorrelated variables such as temperature and air pressure. To date, the effects of low air pressure have been tested especially on human or animal physiology [29] and in the context of space and Mars exploration programs [30, 31]. Additionally, some studies on model plants are conducted in growth chambers [24, 32]. Furthermore, some relevant studies are based only on theoretical assumptions [13, 33] or elevational transect observations [34, 35] as only a few scientifically available hypobaric chambers exist worldwide to test these effects under controlled conditions.

Here, using a unique Ecotron facility, we exposed three plant species with a broad elevation range, *Trifolium pratense*, *Hieracium pilosella*, and *Brachypodium rupestre*, to a reduction in air pressure alone and combined with different water regimes, and we determined their physiological response. We measured changes in stomatal conductance, chlorophyll content, SLA, leaf water content, plant aboveground biomass, and aboveground C and N content of plants that were transplanted into Ecotron chambers for four weeks. By simulating different air pressure scenarios, we aimed to: (i) assess how these plant species—a legume, a forb, and a grass—respond to reduced air pressure in terms of functional traits, water status, and biomass production, and (ii) determine whether variations in irrigation regimes amplify or mitigate the effects of low air pressure on plant performance.

## 2 | Materials and methods

### 2.1 | Experimental setup

Three perennial plant species were chosen for this study: i) a legume (*Trifolium pratense* L.), ii) a forb (*Hieracium pilosella* L.), and iii) a grass (*Brachypodium rupestre* (Host) Roem. & Schult). *T*. *pratense* and *H*. *pilosella* are regularly found between the colline and alpine belt instead, *B*. *rupestre* usually grows in colline-montane belt and irregularly in subalpine one [36]. Plants were selected from a montane grassland (*Festucetum valesiacae*) located at 1,500 m above sea level (a.s.l.) in the Long-Term Socio-Ecological Research (LTSER) site in Matsch/Mazia (South Tyrol, Italy; 46°41’04.2"N, 10°35’08.5"E). Despite their wide elevational range distribution, these species occur frequently in this region. Approximately one month after snowmelt, on May 23^rd^ 2022, 60 individuals (soil cores that hosted multiple individuals) of each species at a similar phenological stage (with unfolded leaves but no visible inflorescence) were collected by extracting plugs (4.8 cm^2^ x 7.5 cm) from the research site at 1,500 m). The plugs were subsequently transplanted into 1.2-L pots (Ø 150 * h 180 mm) filled with sieved soil (4 mm) from the same site where the plants were collected (S1 Fig). The pots were transferred to the terraXcube Ecotron center (https://terraxcube.eurac.edu/) and incubated for a four-week period. The terraXcube is an extreme climate simulation infrastructure capable of controlling crucial abiotic parameters, such as temperature, humidity, solar radiation, and air pressure (see the S1 Table for details). To understand how plants respond to changing air pressure, a gradient design was set up in three 9 m^2^ Ecotron chambers, each simulating different air pressure level: 85 kPa, which corresponds to 1,500 m a.s.l. representing the elevation where target plant species were sampled (control scenario); 75 kPa, corresponding to 2,500 m a.s.l. representing a moderate decrease in atmospheric pressure for plants from 1,500 m a.s.l.; and 62 kPa, corresponding to 4,000 m a.s.l., representing the lowest air pressure that could be simulated in our chambers. The effect of air pressure was disentangled from the effects of other physical parameters that usually co-vary with elevation, particularly temperature, humidity, and solar radiation. The same set of temperature, humidity, and solar radiation was applied in all three chambers, corresponding to the average values recorded in the field by a climate station located at the 1,500 m LTSER site (average values, temperature = 18°C, humidity = 43%, light = 620 μmol/m^2^s^-1^). Temperature, humidity and light were varied hourly to simulate field conditions with the same cycle in all chambers (detailed summary in S2 Table). The chronological trend graph of the temperature and relative humidity process remained stable inside the chambers throughout the 4-weeks testing with continuous monitoring by the chamber control system (S2 Fig). Diurnal light was provided with a cyclic day-night pattern by two LED sensors placed on the ceiling above the plants (S2 Table). Actually, the partial pressure of component gases inside the chambers was not directly controlled. However, the CO_2_ levels were measured, ranging from 350–420 ppm, with brief peaks reaching up to 900 ppm during the measurements. Moreover, no significant increase in CO_2_ was observed outside the building that could have affected our experiment (S3 Fig).

In each chamber, 20 potted individuals of each species were distributed on two benches. Two irrigation systems were installed for each bench to simulate different soil water states in all chambers: (*i*) a higher flow rate (single nozzle flow rate of 6 L/h of H_2_O per pot, wet treatment), and (*ii*) a reduced flow rate (single nozzle flow rate of 2 L/h of H_2_O per pot, dry treatment). The reduced flow rate (dry treatment) simulated the atmospheric, summer field conditions where plants were collected [37], while the higher flow rate (wet treatment) reproduced a continuous water-saturated soil. Moreover, the wet treatment simulated the conditions of fully water-saturated soil which might occur after moderate-heavy rain events. Each pot was irrigated by a single nozzle every 48 h. The wet treatment corresponded to a water supply of 5.7 mm (0.1 L) of water per pot every 48 h which resulted in soil water content between 38 and 45%, and the dry treatment corresponded to a water supply of 1.9 mm (0.033 L) of water per pot every 48 h which resulted in soil water content between 25 and 35%. The target values (25–35% vs. 38–45%) have been measured in the LTER site and the amount of water in the chamber has been set accordingly. Both irrigation systems were coupled with a UV water steriliser. Overall, 180 pots were distributed throughout the three chambers with the following scheme: 5 individuals × 2 water treatments × 2 benches × 3 species × 3 elevations (n = 180) (S4 Fig). Within each bench, the positions of the pots were randomised weekly. However, each pot always experienced the same air pressure and the same water treatment during the experiment. Moreover, the elevation (air pressure) treatment was changed three times between the chambers, with plants following their elevational (air pressure) conditions (each pot was physically translocated from one chamber to another and always experienced the same air pressure conditions). This procedure minimised the limitations of the facility (only one replicate chamber for each air pressure treatment) and reduced the potential variability in microclimatic conditions between the chambers.

### 2.2 | Trait measurements

Seven functional traits were measured on one or more leaves of each plant species depending on the trait: stomatal conductance (gs), chlorophyll content, specific leaf area (SLA), leaf water content (LWC), plant aboveground biomass (phytomass), aboveground biomass C and N content. Leaves were selected excluding the younger and newly developed ones. Gs, chlorophyll content, and SLA, were measured three times to observe the temporal trend of selected parameters: at the start of the experiment (t0, day 0), after two weeks (t1, day 14), and at the end of the experiment, after four weeks (t2, day 30). Due to technical and medical limitations, we restricted the entrance to the chambers to two short access (1 h ca) for rotation (first rotation, between t0 and t1, day 9; second rotation between t1 and t2, day 18) and one long stay (2h per chamber) for the measurements. The gs and the chlorophyll content were measured by selecting one top leaf from a single individual within each pot by using a portable leaf porometer (SC-1, METER Group, Inc., Pullman, WA, USA) and a CCM-300 Chlorophyll Meter (Opti-Sciences, Hudson, NH, USA). Both the instruments were recalibrated whenever they were employed in a different chamber. The SLA was measured according to standard protocols [38]. For the determination of SLA, five fully expanded and photosynthetically active top leaves of *T*. *pratense*, one of *H*. *pilosella*, and two of *B*. *rupestre* were randomly selected from one individual per pot and sampled. The number and morphology of leaves differed between species, therefore, sampling different numbers of leaves per species was necessary to ensure a sufficient number of leaves throughout the sampling period and at the end of the experiment. Leaf area (cm^2^) was estimated by scanning the sampled leaves using ImageJ (http://rsb.info.nih.gov/ij/). The same leaves were weighed (fresh weight, FW) and then oven-dried in a paper bag at 70°C for 72 h to estimate the dry weight (DW) and to calculate the LWC as (FW-DW) / DW. At t2, the aboveground biomass of each pot was collected and oven-dried in a paper bag at 70°C for 72 h to estimate the plant biomass in [g]. Dry aboveground biomass was then used to measure the aboveground biomass C and N content. A CN analyser (CN828, LECO, MI, USA) was used to determine the carbon (C [%]) and nitrogen (N [%]) content in the aboveground biomass. All the previous measurements were conducted before inflorescence appeared.

### 2.3 | Statistical analysis

Data analysis was performed in R 4.3.0 using the R package “lme4” [39]. Before analysis, the relative values in trait values between sampling dates [t0, t1, or t2] were calculated for gs, chlorophyll content, and SLA at the pot level (x), as follows:

$$Relative\;values\;(gs,chlorophyll\;content,SLA)=(x_{t1\;or\;t2}-x_{t0})/(x_{t1\;or\;t2}+x_{t0})$$

reflecting both the extent and direction of the change between t1, t2 and t0. The following analyses refer to the relative values of gs, chlorophyll content, and SLA.

The effect of air pressure and water treatment on plant functional traits was modelled using linear mixed-effects models (LMMs) fit with REML and *t*-tests using Satterthwaite’s method [’lmerModLmerTest’]. The relative values in gs, chlorophyll content, and SLA were modelled as functions of air pressure [P], water treatment [W], time [T], and their interactions. To determine the most appropriate random effect structure, eight models with the following random-effect structures were built considering as random intercepts pot ID, bench, chamber, bench/irrigation, chamber/bench, chamber/irrigation, chamber/bench/irrigation, and bench/irrigation/pot ID respectively. All the factors were nested with 60 levels (replica) for pot ID, 2 levels for irrigation, 2 for bench, and 3 for chamber. The Akaike information criterion (AIC) was used to assess the importance of the different random effects structures [40]. Based on this assessment, only pot ID was retained as a random intercept in all LMMs. The non-significant model parameters were removed using a stepwise selection procedure based on a logarithmic likelihood ratio test with an alpha level and a *p*-value threshold of 0.05 (step.lmerModLmerTest {lmerTest}). We calculated an ANOVA table from the reduced model with *F-tests* and *p-values* using Satterthwaite’s method for the denominator degrees of freedom and *F*-statistic. The model assumptions were validated by examining the normality of the residuals, normality of random effects, homogeneity of variance, and multicollinearity (check_model function in the performance package of R version 4.1.3).

Separately, the effects of [P], [W], and their two-way interactions on LWC, plant aboveground biomass, C content, and N content were also analysed using LMMs and analysis of variance (ANOVA). Due to a non-normal distribution of data, a logarithmic transformation was applied to the aboveground biomass data. A post-hoc pairwise comparison was performed when there was a significant difference between water treatments and air pressure using the emmeans package [41] (emmeans function; degrees-of-freedom method: Kenward–Roger; confidence used: 0.95; p-value adjustment: Tukey method for comparing a family of six estimates; alpha level = 0.05).

## 3 | Results

In this section, we present the results of our study on the impact of low air pressure on *T*. *pratense*, *H*. *pilosella*, and *B*. *rupestre*. First, we describe how these plants respond to reduced air pressure alone in terms of stomatal conductance (gs), chlorophyll content, and SLA. Next, we explore how air pressure might affect survival and growth by analysing resource allocation, and biomass production. Finally, we report if and how the low air pressure combined with different water regimes impacts aforementioned plant parameters. In the last section, we present and discuss the results of the combined effect only when it was statistically significant.

### 3.1 | Effects of low air pressure on gs, chlorophyll content and SLA

**Fig 1:**
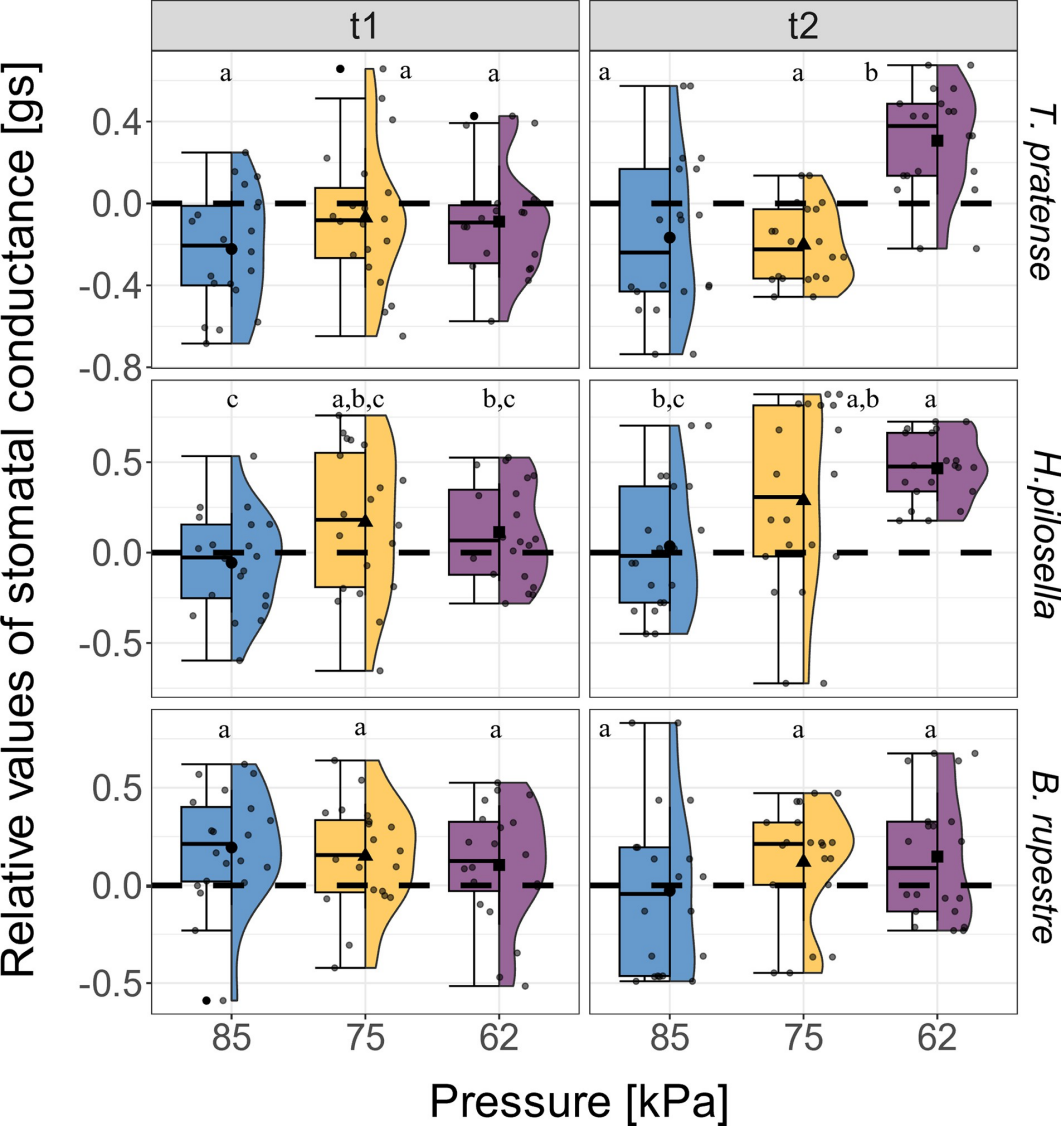
Effect of low air pressure on the relative values of stomatal conductance [gs]. Effect of pressure [kPa] and time (t1 and t2) on the relative values of stomatal conductance [gs] in *Trifolium pratense* (n = 20), *Hieracium pilosella* (n = 20) and *Brachypodium rupestre* (n = 20). Relative values for t1 and t2 are compared to t0 (black dotted line). The blue, yellow, and purple boxplot indicate the three air pressures tested in the chambers (85, 75, and 62 kPa). Lowercase letters indicate significant differences according to the post-hoc test comparison (*p* < 0.05). Whiskers extend to the minimum and maximum values within 1.5 times the IQR from Q1 and Q3, respectively. Dots out of the whisker interval indicate outliers. See S3 Table for the mean values in [mmol m^-2^ s^-1^] ± sd.

Our results showed a significant effect of low air pressure on gs of *T*. *pratense* at t2, which exhibited higher values at 62 kPa (Fig 1). Notably, the same increasing trend is observable also in *H*. *pilosella* (Fig 1), but there were no significant pressure related variations in *B*. *rupestre* (Fig 1).

**Fig 2:**
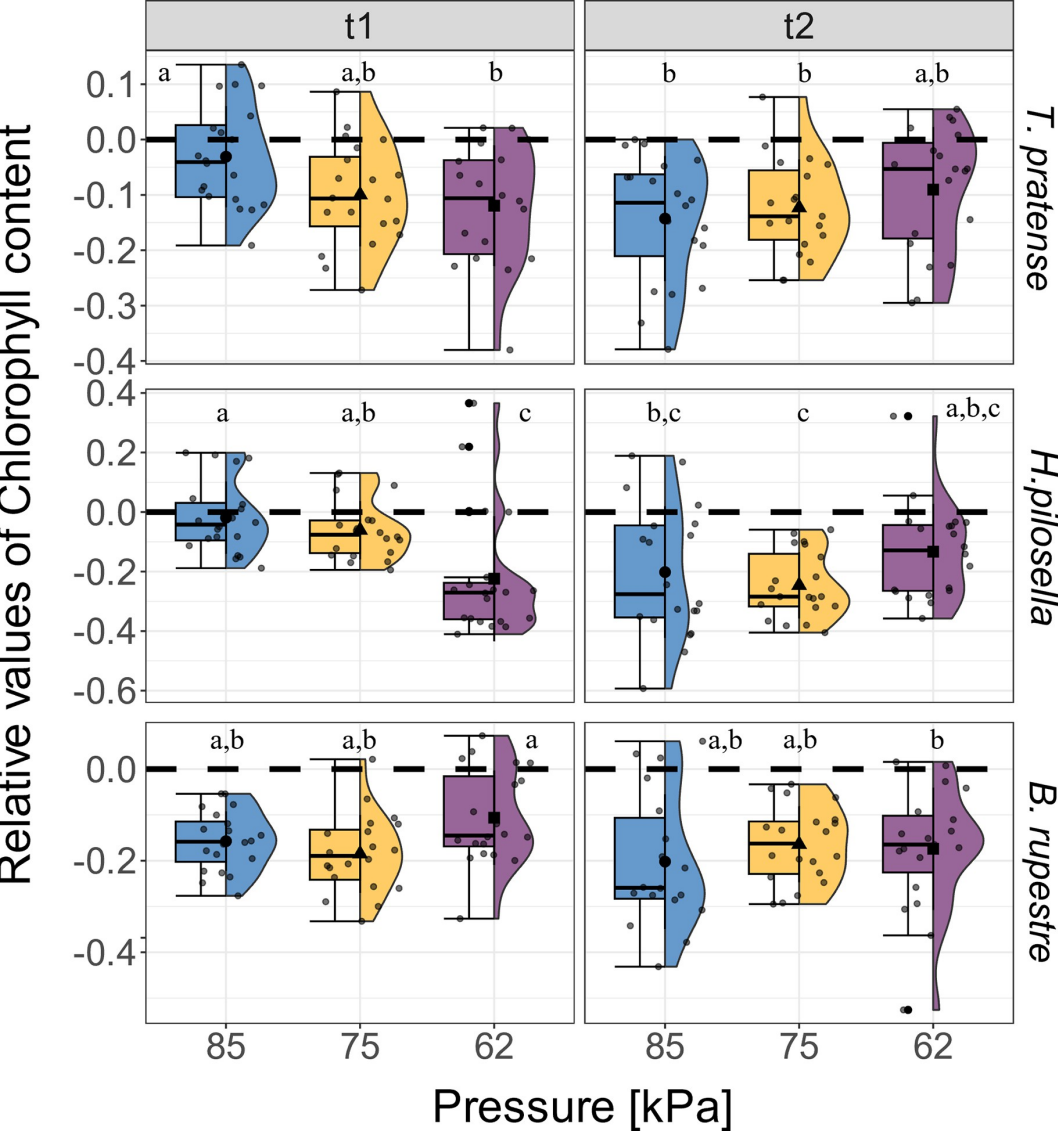
Effect of low air pressure on the relative values of chlorophyll content. Effect of pressure [kPa] and time (t1 and t2) on the relative values of Chlorophyll content in *Trifolium pratense* (n = 20), *Hieracium pilosella* (n = 20) and *Brachypodium rupestre* (n = 20). Relative values for t1 and t2 are compared to t0 (black dotted line). The blue, yellow, and purple boxplot indicate the three air pressures tested in the chambers (85, 75, and 62 kPa). Lowercase letters indicate significant differences according to the post-hoc test comparison (p < 0.05). Whiskers extend to the minimum and maximum values within 1.5 times the IQR from Q1 and Q3, respectively. Dots out of the whisker interval indicate outliers. See S4 Table for the mean values in [mg m^-2^] ± sd.

Conversely, low air pressure significantly diminished relative values of chlorophyll content in *T*. *pratense* and *H*. *pilosella* (Table 1), with the lowest values observed at t1 and at 62 kPa (Fig 2). However, at t2, the relative chlorophyll content was similar at all air pressures for the above-mentioned species. Then, low air pressure did not significantly affect chlorophyll content in *B*. *rupestre* (Table 1 and Fig 2).

**Table 1:** Impact of pressure, water, and time on leaf traits.

Species	Treatment	gs		chlorophyll content		SLA	
		*F*	*P*	*F*	*P*	*F*	*P*
*Trifolium pratense*	P × W		ns	4.754	0.013	4.754	0.013
	P × T	13.654	<0.001	12.075	<0.001	12.075	<0.001
	W × T		ns	15.953	<0.001	10.239	0.002
*Hieracium pilosella*	P	5.846	0.005		ns	4.922	ns
	P × T			22.722	<0.001		0.011
*Brachypodium rupestre*	W × T		ns		ns	6.330	0.015
	P × W × T		ns	5.138	0.009		ns

Effects of pressure [P], water [W], time [T], and their interactions on stomatal conductance (gs, n = 10), chlorophyll content (n = 10) and specific leaf area (SLA) of *Trifolium pratense* (five leaves per sample, n = 50), *Hieracium pilosella* (one leaf per sample, n = 10) and *Brachypodium rupestre* (two leaves per sample, n = 20).

Note: This table summarises the outputs of the linear mixed models (Type III Analysis of Variance table with Satterthwaite’s method) after a stepwise selection procedure. Only significant interactions (p < 0.05) are reported.

**Fig 3:**
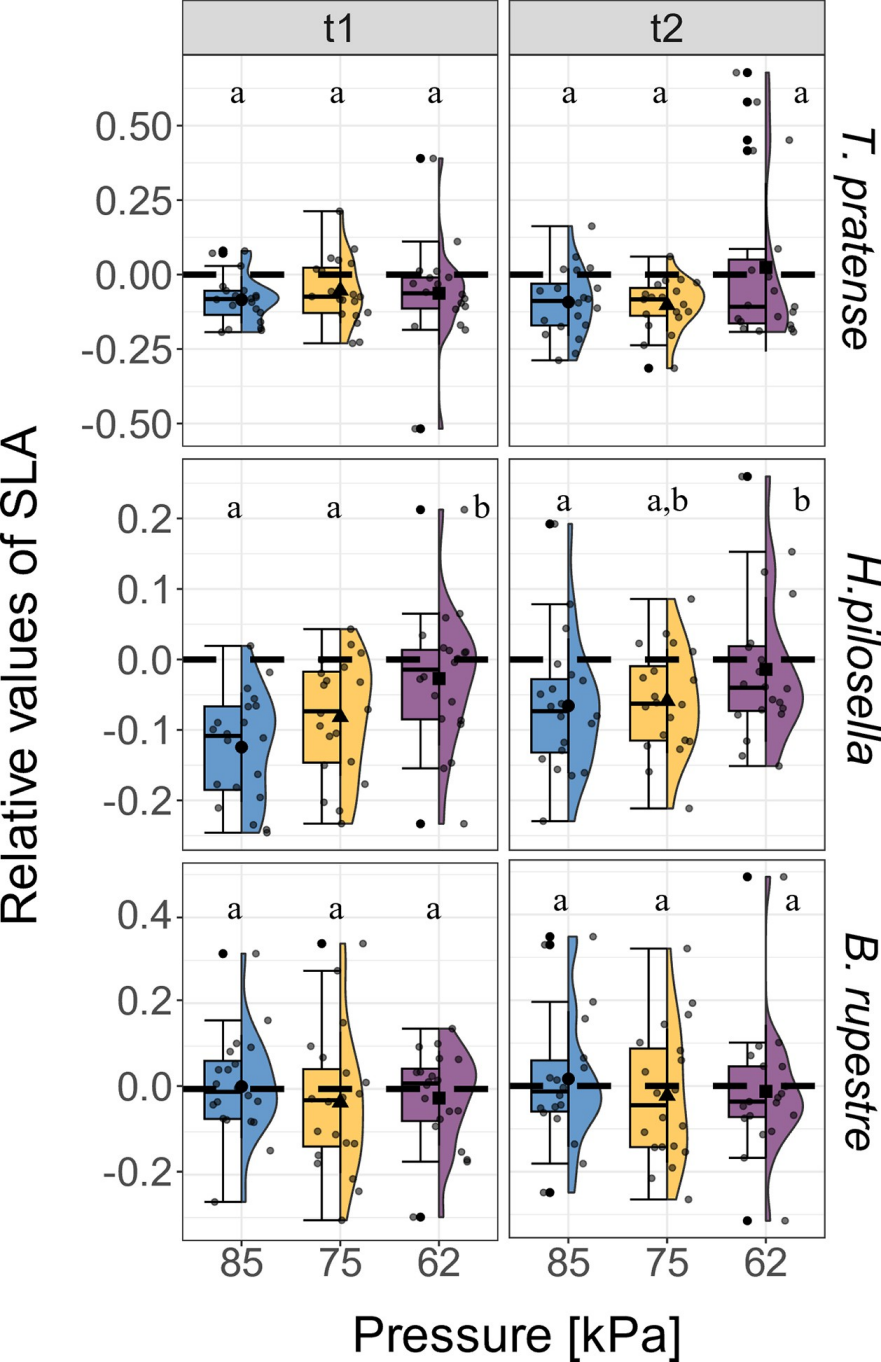
Effect of low air pressure on the relative values of Specific Leaf Area [SLA]. Effects of pressure [kPa] and time (t1 and t2) on relative values of SLA in *Trifolium pratense* (n = 20), *Hieracium pilosella* (n = 20) and *Brachypodium rupestre* (n = 20). The blue, yellow, and purple boxplot indicate the three air pressures tested in the chambers (85, 75, and 62 kPa). Lowercase indicate significant differences according to the post-hoc test comparison (*p* < 0.05). Whiskers extend to the minimum and maximum values within 1.5 times the IQR from Q1 and Q3, respectively. Dots out of the whisker interval indicate outliers. See S5 Table for the mean values in [cm^*2*^ g^*-1*^].

SLA was significantly higher at 62 kPa only in *H*. *pilosella* (Fig 3), while no significant pressure-related variations were observed for the other two species (Fig 3).

### 3.2 | Effects of low air pressure on resource allocation and plant aboveground biomass

**Fig 4:**
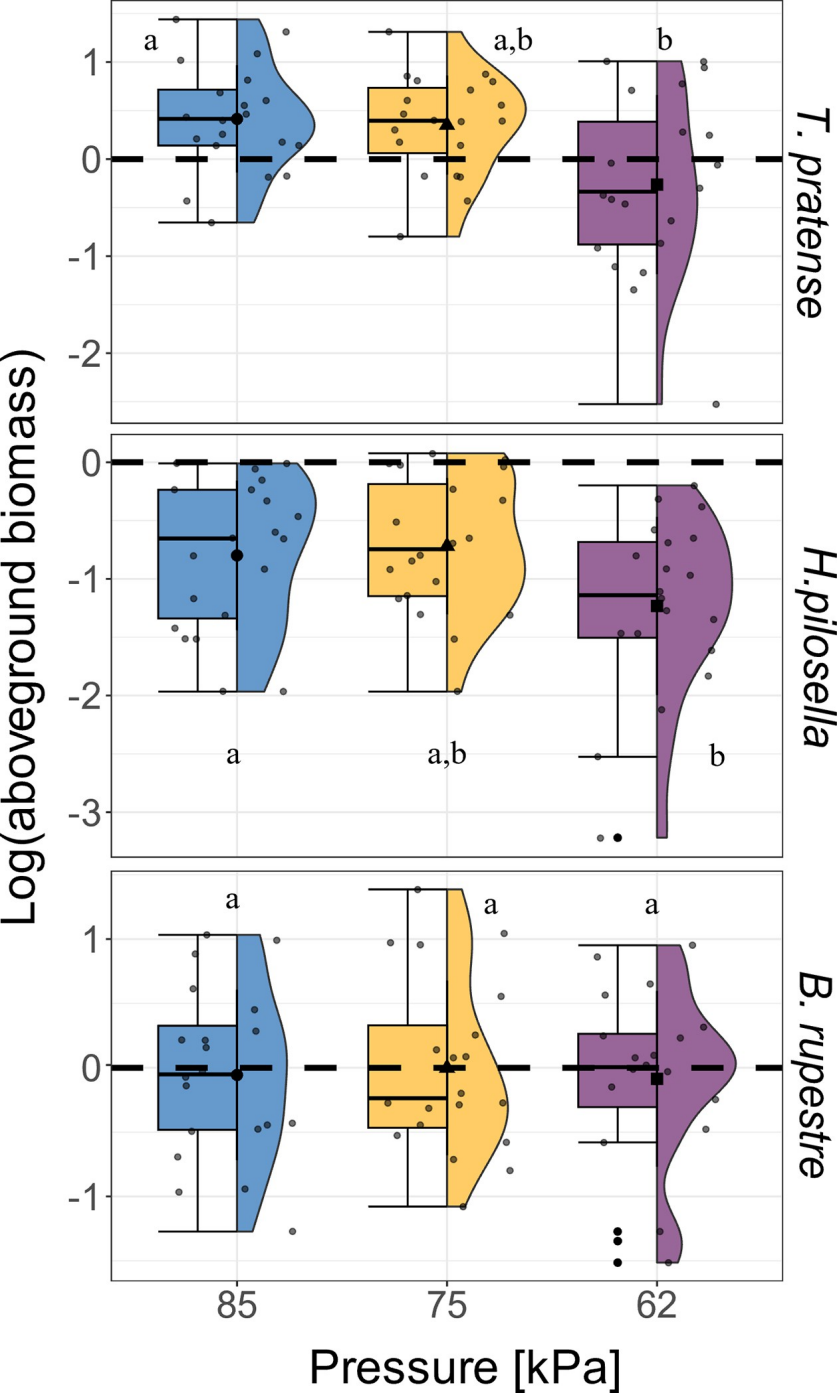
Effect of low air pressure on aboveground biomass. Effects of pressure [kPa] on log(aboveground biomass) in (a) Trifolium pratense (n = 20), (b) Hieracium pilosella (n = 20) and (c) Brachypodium rupestre (n = 20) at t2. The blue, yellow, and purple boxplot indicate the three air pressures tested in the chambers (85, 75, and 62 kPa). Lowercase letters indicate significant differences according to the post-hoc test comparison (p < 0.05). Whiskers extend to the minimum and maximum values within 1.5 times the IQR from Q1 and Q3, respectively. Dots out of the whisker interval indicate outliers.

**Fig 5:**
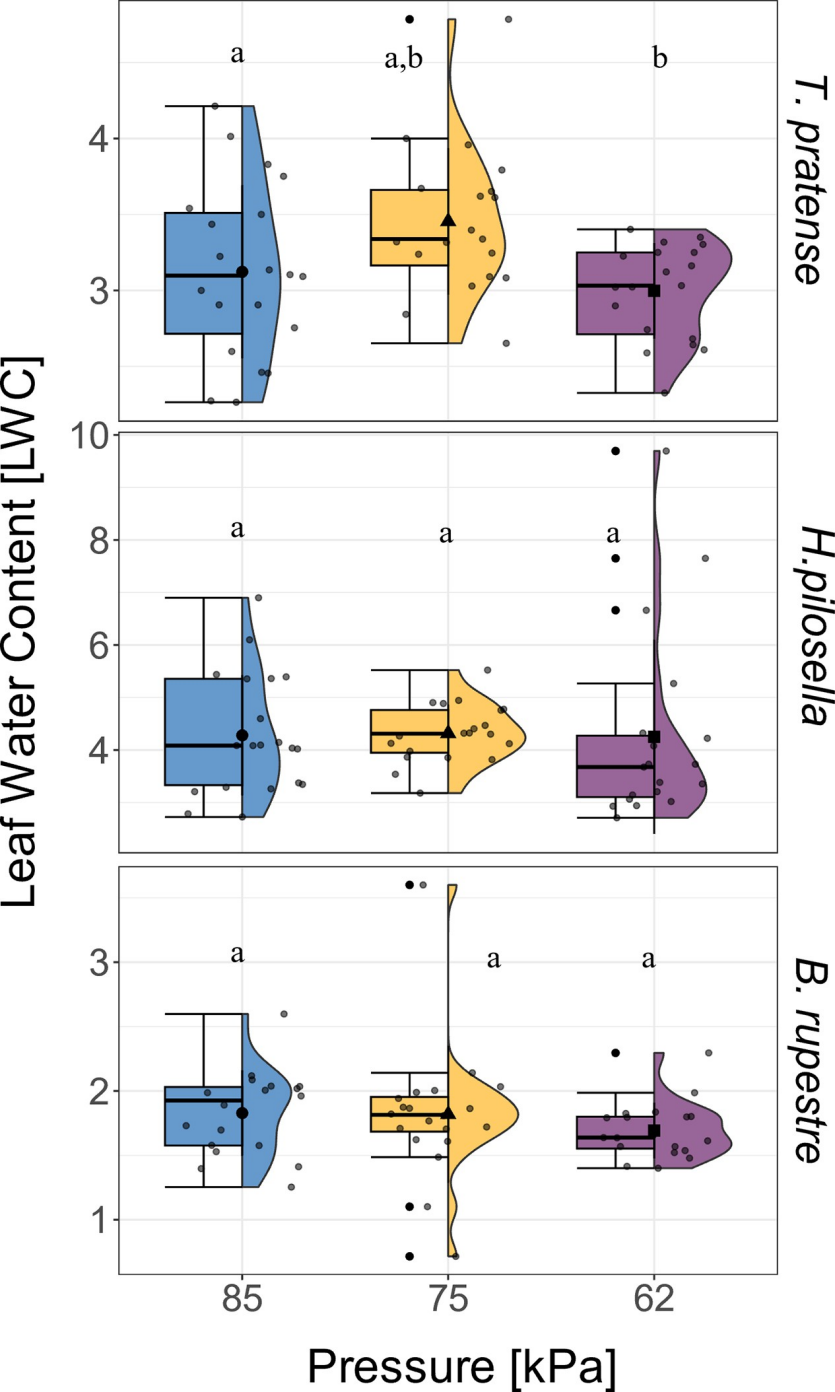
Effect of low air pressure on Leaf Water Content [LWC]. Effects of pressure [kPa] on Leaf Water Content [LWC] in (a) *Trifolium pratense* (*n* = 20), (b) *Hieracium pilosella* (*n* = 20) and (c) *Brachypodium rupestre* (*n* = 20) at t2. The blue, yellow, and purple boxplot indicate the three air pressures tested in the chambers (85, 75, and 62 kPa). Lowercase letters indicate significant differences according to the post-hoc test comparison (*p* < 0.05). Whiskers extend to the minimum and maximum values within 1.5 times the IQR from Q1 and Q3, respectively. Dots out of the whisker interval indicate outliers.

At t2, the aboveground biomass of *T*. *pratense* and *H*. *pilosella* was lower at 62 kPa (Fig 4). Similarly, the percentage of C in aboveground biomass was reduced by 3% in the case of *T*. *pratense* and by 5% in the case of *H*. *pilosella* at 62 kPa compared to 85 kPa (S5 Fig). However, no significant changes related to low air pressure were observed for either for aboveground biomass (Fig 4) or C content in aboveground biomass (S5 Fig) in *B*. *rupestre*. Low air pressure also decreased the LWC in *T*. *pratense* (Table 2), which was lower at 62 kPa compared to 85 kPa and 75 kPa (Fig 5). However, no significant variations were observed for the other two species (Fig 5).

**Table 2:** Impact of pressure and water on aboveground biomass composition.

Species	Treatment	C content		N content		LWC		Aboveground biomass	
		*F*	*P*	*F*	*P*	*F*	*P*	*F*	*P*
*Brachypodium rupestre*	P	3.163	0.050	5.695	0.006			0.081	
	W			4.629	0.036			1.833	
	P × W			6.721	0.002			0.509	
*Trifolium pratense*	P	10.248	< 0.001			4.773	0.012	7.018	0.002
	W			17.678	< 0.001			8.088	0.006
	P × W	3.549	0.034					2.799	
*Hieracium pilosella*	P	7.548	0.001					3.347	0.043
	W							1.211	
	P × W	5.261	0.008					0.233	

Effects of pressure [P], water [W] and their interactions on aboveground biomass C content (C content), aboveground biomass N content (N content), leaf water content (LWC), and aboveground biomass.

*Note*: This table summarises the results of the ANOVA. Only significant terms were reported in the table after a stepwise selection procedure (*p* < 0.05). Aboveground biomass was logarithmically transformed. *n* = 20 plants per species.

### 3.3 | Combined effects of low air pressure and different water supply

**Fig 6:**
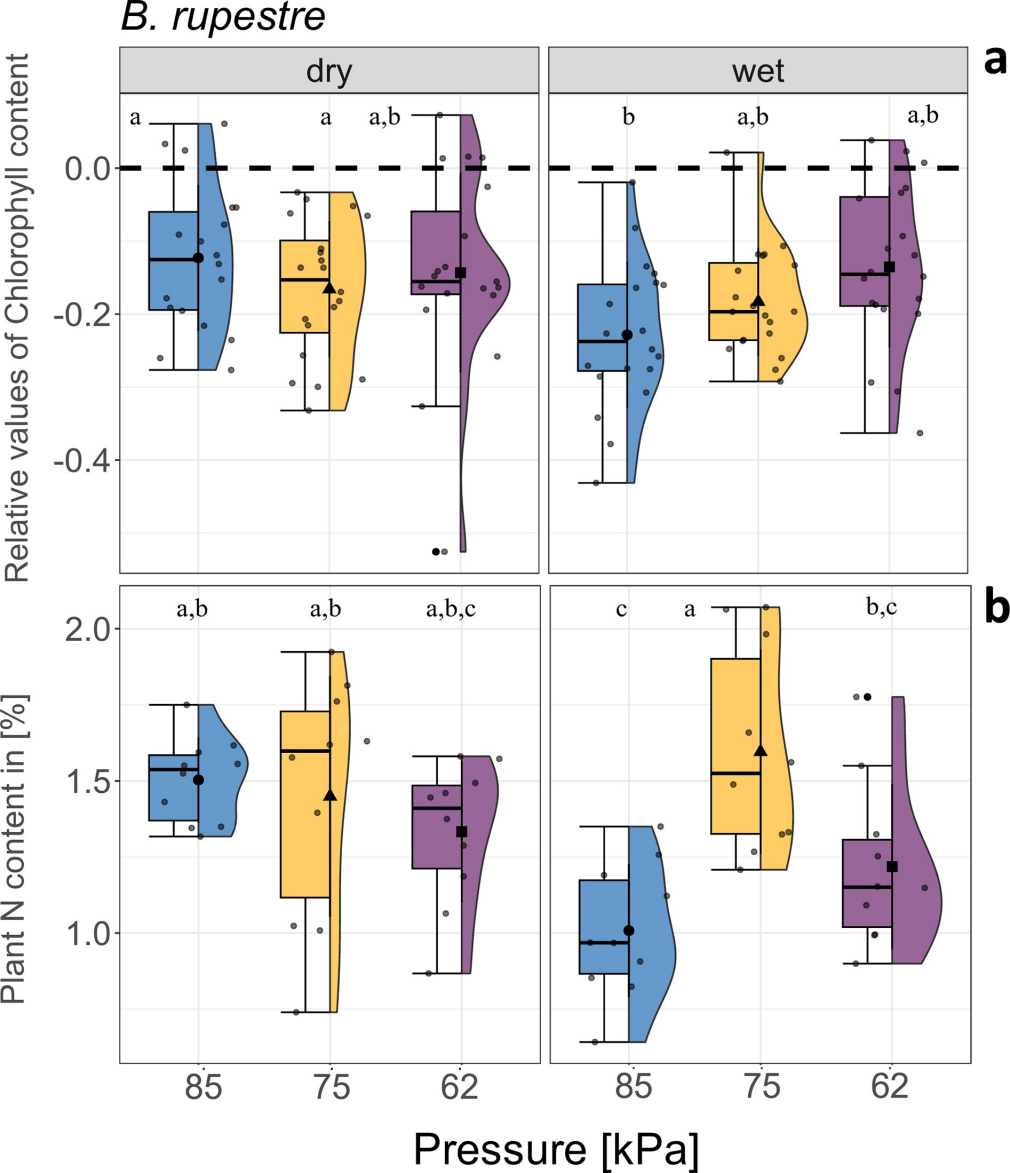
Combined low air pressure and water treatment effect on chlorophyll and nitrogen content. Interactive effects of pressure [kPa] and water treatment on ***(a)*** chlorophyll content (n = 20) and ***(b)*** N content in aboveground biomass (n = 10) of *Brachypodium rupestre*, at t2. The blue, yellow, and purple boxplot indicate the three different air pressures tested in the chambers (85, 75, and 62 kPa). Lowercase letters indicate significant differences according to the post-hoc test comparison (p < 0.05). Whiskers extend to the minimum and maximum values within 1.5 times the IQR from Q1 and Q3, respectively. Dots out of the whisker interval indicate outliers. See S6 Table for the mean values of chlorophyll content in [mg m^-2^] ± sd.

When considering the combined effect of air pressure and water availability ([P] x [W]), the ANOVA test showed no significant effect in gs, C content, and aboveground biomass in any of the three considered species (Table 1). However, relative chlorophyll content and N content of aboveground biomass were influenced by the interactive effects of [P] × [W], in case of *B*. *rupestre* (Table 1). Specifically, after four weeks of incubation, the chlorophyll content was significantly diminished at 85 kPa under higher water soil conditions (wet) compared with lower soil water conditions (dry) (Fig 6A). A similar trend was also observable for nitrogen content (Fig 6B). Nevertheless, we observed no significant difference in these parameters for *T*. *pratense* and *H*. *pilosella* (S6 Fig) were established after four weeks of incubation. Additionally, the ANOVA test revealed a significant interaction effect of [W] x [T] on SLA in both *T*. *pratense* and *B*. *rupestre* (Table 1) nevertheless, pairwise comparison did not show any significant differences between the water treatments for either species (S7 Fig).

## 4 | Discussion

With ongoing climate change, most organisms are expected to shift upward to track their thermal niches, testing novel environmental conditions such as a reduction in air pressure which reduces the partial pressure and enhances the diffusivity of atmospheric gases [13]. The complexity of simulating different atmospheric conditions necessitates appropriate experimental facilities and setups [42]. Our experiment employed such a facility, allowing us to focus on the mechanisms underlying plant responses to lower air pressures. In this study, we exposed plant-soil mesocosms to air pressures of 85 kPa (corresponding to 1,500 m a.s.l.), 75 kPa (corresponding to 2,500 m a.s.l.), and 62 kPa (corresponding to 4,000 m a.s.l.). We observed a decrease in relative chlorophyll content after two weeks and in aboveground biomass production after four weeks in both *T*. *pratense* and *H*. *pilosella*. In these two species, we also observed a higher stomatal conductance at 62 kPa after four weeks. Additionally, we found a decrease in chlorophyll and N content at 85 kPa under wet conditions for *B*. *rupestre*. However, a reduced water supply regime appeared to mitigate the effect of low air pressure on both these parameters.

### 4.1 | Effect of reduced air pressure on chlorophyll content

After two weeks of exposure to low air pressure, *T*. *pratense* and *H*. *pilosella* plants showed a diminished chlorophyll content at 62 kPa. Chlorophyll content in plants is mainly related to light irradiance and photon flux density [14]; however, in our experiment, all plants were arranged on a table side-by-side basis and exposed to a perpendicular light source (with the same cycle of light intensities in all chambers). Therefore, as all plants we sampled were at a similar phenological stage and from the same atmospheric pressure, our results suggest that the differences in chlorophyll content might be due to low air pressure rather than light. However, at 62 kPa after four weeks, the chlorophyll content in *T*. *pratense* and *H*. *pilosella* plants exhibited no differences compared with the other two air pressures. This behaviour may be interpreted as an acclimatization to reduced total air pressure and decreased partial pressure of atmospheric gases. Thus, lower air pressure might be considered a new environmental scenario with which plants have to cope, including compensating for the lesser partial pressure of gases, especially CO_2_ [7], to survive at higher elevations [14, 43].

### 4.2 | Effect of reduced air pressure on leaf functional traits and biomass production

As already documented by Körner, 2021 [7], plants shrink SLA along elevational gradients to maximize the efficiency of photosynthetic capacity: by minimising leaf area and enhancing the thickness of the palisade mesophyll, they reduce leaf gas diffusivity while enhancing the content of photosynthetic enzymes per area, leading to more efficient C fixation per unit area. Conversely, some plants can also enlarge SLA to maintain photosynthetic efficiency at higher elevations, effectively capturing light [44], as observed in *H*. *pilosella* at 62 kPa, which corresponds to 4,000 m a.s.l.. However, *T*. *pratense* and *B*. *rupestre* did not show any effect of low air pressure on SLA. The lack of a clear effect could be due to the phenotypic character of SLA. Since the trait is mainly related to temperature and season length, in addition to leaf ontogeny [45], these factors could be masking any effect of reduced air pressure.

Low air pressure, though, rises gas diffusivity leading to an increase in leaf transpiration, as suggested by Gale in 1972 [46]. In our study, we observed a higher stomatal conductance in both *T*. *pratense* and *H*. *pilosella* at 62 kPa, but a decreased leaf water content only in *T*. *pratense* between 75 and 62 kPa. Instead, *B*. *rupestre* and *H*. *pilosella* showed no significant difference in leaf water content. This can be explained by the ecological requirements of *B*. *rupestre* and *H*. *pilosella*, considered drought-tolerant species that favour dry soils, and those of *T*.*pratense*, which requires mesic environments [47] Indeed, vapor loss from plants was already reported to strongly depend on vegetation type and the inherent differences in water requirements, e.g. [48].

On the other hand, the shoot biomass and the aboveground C content significantly lessened in response to the low air pressure in both species, *T*. *pratense* and *H*. *pilosella*. This reduction might be related to a reallocation of resources for maintenance and likely to the growth of the underground biomass. This would be in line with a study that has reported a significant reduction in growth and different morphogenetic responses induced by low air pressure treatments [49], particularly in root and leaf growth. However, this morphogenetic response may be less manifested over a short-period experiment in slow-growing species such as *B*. *rupestre* [50], where we did not find differences between air pressures during our experiment.

Nevertheless, another aspect to consider when observing the physiological response of plants exposed to *in vitro* conditions is that plants are suddenly exposed to new environmental conditions that they must cope with, whereas *in situ* experiments, plants are already adapted to local environmental conditions. So, in this respect, growth cessation or decrease might be interpreted as a sign of stress [51].

### 4.3 | Differential water supply effect on plant response to low air pressure

Our findings did not reveal any notable differences in chlorophyll content or aboveground nitrogen (N) content of *T*. *pratense* and *H*. *pilosella* plants at 75 and 62 kPa under different water availability regimes; however, the chlorophyll content in *B*. *rupestre*, though, was significantly lower under the wet treatment at 85 kPa. This decrease could be attributed to the significant reduction in N content in the aboveground biomass observed under the same conditions. However, the effects of the different water treatments were not observed within the different air pressures.

We expected an increase in SLA with a diminished level of water supply as higher SLA is often associated with elevated temperatures and lower soil water content [52]. However, SLA did not vary significantly between the water treatments for either *B*. *rupestre* or *T*. *pratense*. Generally, grasses have great phenotypic plasticity to tolerate the harsh environmental conditions typical of high-elevation ecosystems [53, 54]. As a grass species, *B*. *rupestre* may behave differently to compensate for the low air pressure effect on N content, thus maintaining a similar chlorophyll content in well-watered plants across the elevational gradient. We might assume that the role of water relations in plants diminishes with increasing elevation as already suggested by previous works [7, 55].

### 4.4 | Limitations and further steps

Further analyses are needed to clarify the effects of low air pressure on plants. In addition to the temperature and atmospheric pressure, other factors vary, such as the partial pressure and availability of O_2_ and CO_2_ [7, 13]. The effects of these factors on plant growth and performance need to be investigated. In conditions of reduced air pressure, the mole fractions of CO_2_ and O_2_ are almost constant, whereas their partial pressures decrease [14] with consequences for organisms that require further investigation. For example, in plants, this diminished partial pressure of O_2_ can lead to a reduced photorespiratory burden in the leaves, which counterbalances the direct effect of a lower CO_2_ concentration on photosynthesis [43]. Furthermore, the difference between saturation and actual water vapor pressure, and thus the vapor pressure deficit, increases with lower air pressure, raising the evapotranspiration rates and the soil water loss [56, 57]. Regarding this aspect, this experimental design did not account for the belowground responses, which could provide a better overview of the different plant investment strategies for balancing growth and adaptation.

Another aspect that needs to be considered is the duration of the experiment. The experiment was limited to four weeks due to the substantial personnel, technical, and financial resources required for such infrastructure. The short duration does not allow us to investigate long-term adaptation. Moreover, our target plants were collected from a field where they were adapted to pre-determined environmental conditions. Thus, the consequences of lower air pressure may be more pronounced after longer treatment periods. Therefore, specific long-term studies, physiological and biochemical analyses will contribute to a better understanding of the responses observed in the present study. Finally, a direct comparison of some aspects of the Ecotron experiments with *in vivo* observations might be helpful in the interpretation of the results and in extrapolating whether the reduced air pressure is impacting plant physiology also in nature.

## 5 | Conclusions

To the best of our knowledge, this is the first study to isolate the effects of air pressure from temperature, relative humidity, and solar radiation on plant physiology. Thus, we explored the possible effects of low atmospheric pressure on plants using an innovative Ecotron facility. In a controlled experiment focused on varying this single parameter, reduced air pressure appears to increase stomatal conductance, hamper aboveground biomass plant production, and directly influence the maintenance of the chlorophyll content at level similar to those under higher air pressure; however, the response is species-specific. The species-specific responses of plants to reduced total atmospheric pressure may potentially explain part of their ability to cope with the varying environmental conditions found at different elevations, as well as their differential responses to climate change-related processes, such as upward migration in mountain areas. This study is unique in that the Ecotron chambers allow for the precise control of air pressure alone; however, some aspects remain unclear, such as the specific effect of the individual gaseous components, and particularly O2 and VPD, as well as the role of belowground interactions on plants.

In conclusion, while environmental parameters (i.e. temperature, irradiation, soil features, biotic factors) interact and influence one other, these initial results suggest that low air pressure plays a significant role in affecting plant performance and requiring adaptive responses. Thus, low air pressure should be included into future experiments aimed at assessing plant responses at higher elevations.

## Supporting information

S1 Fig Plant status. Transplanted *Trifolium pratense* and *Hieracium pilosella* plants in 1.2 L pots after the sampling in Mazia Valley. All the plants were collected at a similar phenological stage (unfolded leaves but no visible inflorescence), on 23^rd^ of May. Plants were selected from the same site (LTSER site, 46°41’04.2"N, 10°35’08.5"E) with similar exposure, slope and bedrock.
(DOCX)

S2 Fig Process of temperature and relative humidity (rH). The following figures show the process of **(a)** temperature in [°C] and **(b)** relative humidity (rH) in [%] over the 4 weeks of testing. SEC1, SEC2 and SEC3 refers to the three 3 × 3 m^2^ chambers, each representing a different elevation (1,500, 2,500, and 4,000 m) and air pressure (85, 75, and 62 kPa). The graphs show the temperature and rH trends inside each chamber from the t0 (26.05) to the t2 (22.06).
(DOCX)

S3 Fig CO2 variation in the chambers. CO2 concentration in [ppm] inside the chambers (SEC1, *blue* line SEC2, *orange* line and SEC3, *green* line) from the t0 (26.05) to the t2 (22.06).
(DOCX)

S4 Fig Schematic design representation. The scheme illustrates the design of each chamber. In each chamber, 60 pots were distributed across two benches. Each bench had five replicates per condition (five individuals × two water treatments × three species, n = 30). Light blue dots represent the wet treatment (0.1 L per pot each 48 h), and red dots represent the dry treatment (0.033 L per pot each 48 h).
(DOCX)

S5 Fig Effect of low air pressure on aboveground C content. Effects of elevation on C content in aboveground biomass of *Trifolium pratense* (*n* = 20), *Hieracium pilosella* (*n* = 20), and *Brachypodium rupestre* (*n* = 20). The blue, yellow, and purple boxplot indicate the three air pressures tested in the chambers (85, 75, and 62 kPa). Lowercase letters indicate significant differences according to the post hoc test comparison (p < 0.05). Dots out of the whisker interval represent outliers.
(DOCX)

S6 Fig Combined low air pressure and water treatment effect on chlorophyll and nitrogen content in *T*. *pratense* and *H*. *pilosella*. Interactive effects of pressure [kPa] and water treatment on ***(a)*** chlorophyll content (n = 20) and ***(b)*** N content in aboveground biomass (n = 10) of *T*. *pratense* and *H*. *pilosella*, at t2. The blue, yellow, and purple boxplot indicate the three different air pressures tested in the chambers (85, 75, and 62 kPa). Lowercase letters indicate significant differences according to the post hoc test comparison (p < 0.05). Dots out of the whisker interval represent outliers.
(DOCX)

S7 Fig Effects of water treatment on SLA in (a) *Trifolium pratense* and (b) *Brachypodium rupestre*. (c) Mean values of specific leaf area (SLA). The interactive effect of water treatment (dry and wet) and time on SLA in ***(a)*** *Trifolium pratense* (*n* = 20) and ***(b)*** *Brachypodium rupestre* (*n* = 20). Relative values for t1 and t2 compared to t0 (black dotted line) are reported in the figures. Lowercase letters indicate significant differences according to the post hoc test comparison (*p* < 0.05). Dots out of the whisker interval represent outliers. (c) Mean values of SLA in [cm^2^ g^-1^] ± sd.
(DOCX)

S1 Table Technical sheet, SMALL CUBE. The SMALL CUBE *(internal dimensions*, *2*.*8 × 3 × 2*.*8 m (L × W × H))* consists of three smaller simulation chambers that can independently replicate the different environmental conditions present in the Alpine region. Three independent chambers were accessible via a common airlock. All environmental parameters were combined simultaneously to simulate complex scenarios. In this table, we show the achievable range for each parameter from minimum to maximum. The structure is also equipped with laboratories for sample preparation and analysis. (*source*: Structure—terraXcube (eurac.edu)).
(DOCX)

S2 Table Test report. Daily cycle of temperature (T), relative humidity (Rh), vapor pressure deficit (VPD), light intensity (light), and photosynthetic photon flux density (PPFD) at hourly intervals, in three Ecotron chambers. PPFD values are measured by a light sensor directly in all three chambers. The elevation (air pressure) treatment was exchanged between the three chambers every 7 days clockwise.
(DOCX)

S3 Table Mean values of stomatal conductance. Mean values in [mmol m^-2^ s^-1^] ± sd of stomatal conductance for *Trifolium pratense* (*n* = 20), *Hieracium pilosella* (*n* = 20) and *Brachypodium rupestre* (*n* = 20) at the beginning (*t*_*0*_), after two (*t*_*1*_) and four (*t*_*2*_) weeks since the start of the experiment at 85, 75, and 62 kPa.
(DOCX)

S4 Table Mean values of chlorophyll content. Mean values in [mg m^2^] ± sd of chlorophyll content for *Trifolium pratense* (*n* = 20), *Hieracium pilosella* (*n* = 20) *and Brachypodium rupestre* (*n* = 20) at the beginning (*t*_*0*_), after two (*t*_*1*_) and four (*t*_*2*_) weeks since the start of the experiment at 85, 75, and 62 kPa.
(DOCX)

S5 Table Mean values of specific leaf area (SLA). Mean values of specific leaf area (SLA) in [cm^2^ g^-1^] ± sd for *Trifolium pratense*, *Hieracium pilosella* and *Brachypodium rupestre* at the beginning (*t*_*0*_), after two (*t*_*1*_) and four (*t*_*2*_) weeks since the start of the experiment at 85, 75, and 62 kPa. Measurements refer to five leaves per sample in *T*. *pratense* (*n* = 20), one leaf per sample in H. pilosella (*n* = 20) and two leaves per sample in *B*. *rupestre* (*n* = 20).
(DOCX)

S6 Table Mean values of chlorophyll content for different water treatment. Mean values in [mg m^2^] ± sd of chlorophyll content for *Brachypodium rupestre* (*n* = 20) at the beginning (*t*_*0*_) and after four weeks (*t*_*2*_) since the start of the experiment at 85, 75, and 62 kPa in dry (d) and wet (w) treatments.
(DOCX)
