# Supplementary material for: Short-term impact of low air pressure on plants’ functional traits
Source: PLoS One. 2025 Jan 15;20(1):e0317590. doi: 10.1371/journal.pone.0317590 (PMC11734969; doi:10.1371/journal.pone.0317590)
Supplement: S1 Fig — Transplanted Trifolium pratense and Hieracium pilosella plants in 1.2 L pots after the sampling in Mazia Valley. All the plants were collected at a similar phenological stage (unfolded leaves but no visible inflorescence), on 23rd of May. Plants were selected from the same site (LTSER site, 46°41’04.2"N, 10°35’08.5"E) with similar exposure, slope and bedrock. (DOCX) [file pone.0317590.s001.docx]

**S1 Fig. Plant status.** Transplanted *Trifolium pratense* and *Hieracium pilosella* plants in 1.2 L pots (Ø 150 * h 180 mm) after the sampling in Mazia Valley. All the plants were collected at a similar phenological stage (unfolded leaves but no visible inflorescence), on 23^rd^ of May. Plants were selected from the same site (LTSER site, 46°41'04.2"N, 10°35'08.5"E) with similar exposure, slope and bedrock.

**
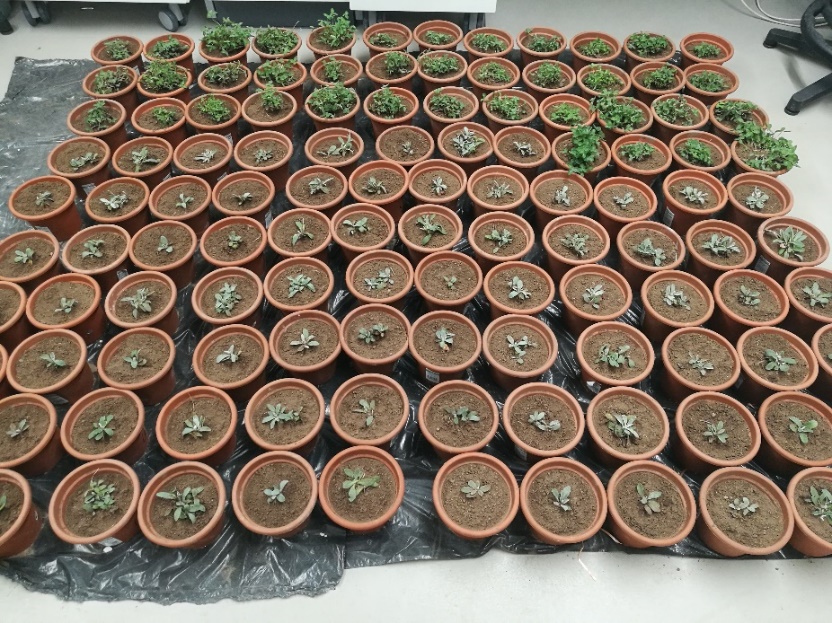
**

*
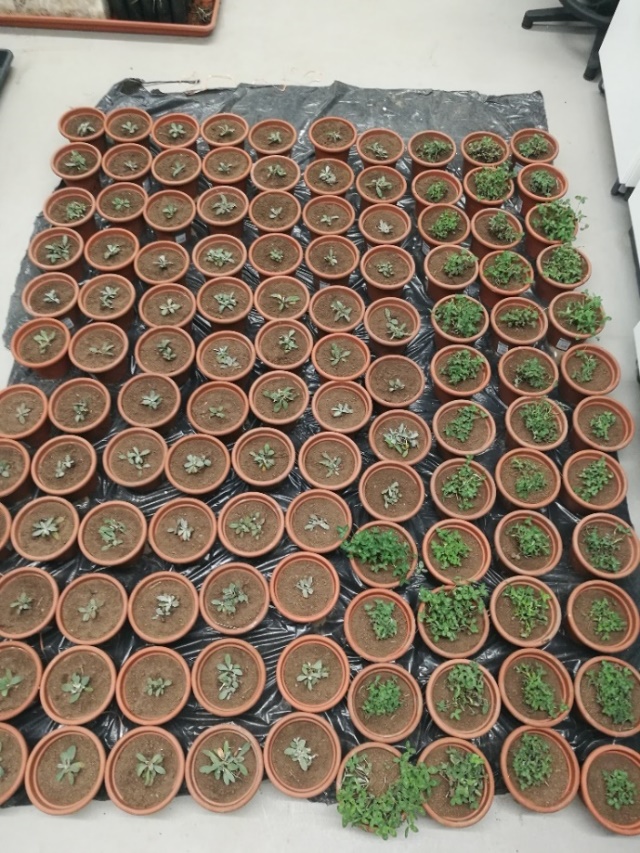
*
