# Supplementary material for: Short-term impact of low air pressure on plants’ functional traits
Source: PLoS One. 2025 Jan 15;20(1):e0317590. doi: 10.1371/journal.pone.0317590 (PMC11734969; doi:10.1371/journal.pone.0317590)

**S2 Fig. Process of temperature and relative humidity (rH).** The following figures show the process of **(a)** temperature in [°C] and **(b)** relative humidity (rH) in [%] over the 4 weeks of testing. SEC1, SEC2 and SEC3 refers to the three 3 × 3 m^2^ chambers, each representing a different elevation (1,500, 2,500, and 4,000 m) and air pressure (85, 75, and 62 kPa). The graphs show the temperature and rH trends inside each chamber from the t0 (26.05) to the t2 (22.06).


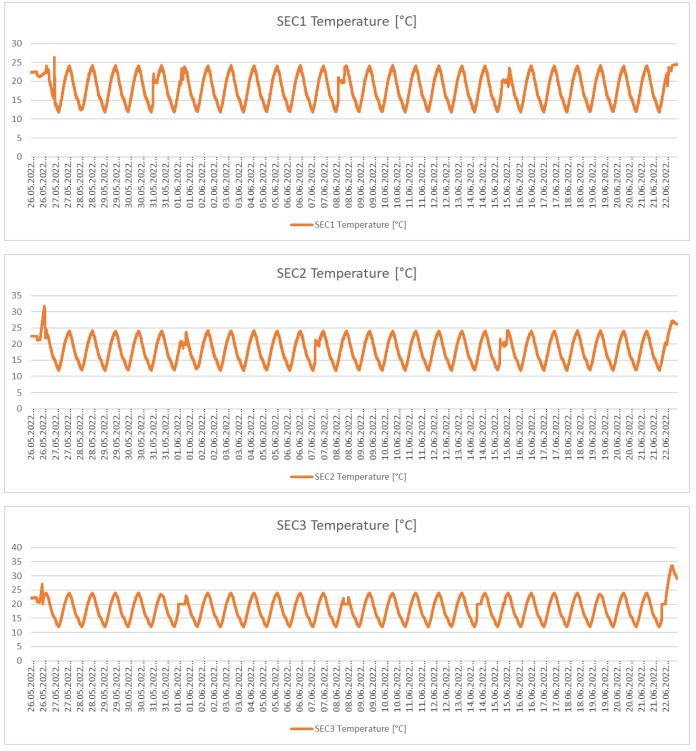
**a**

**b**


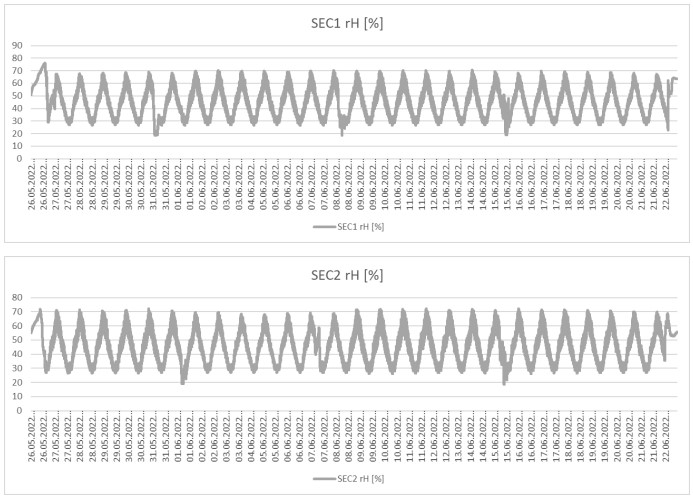

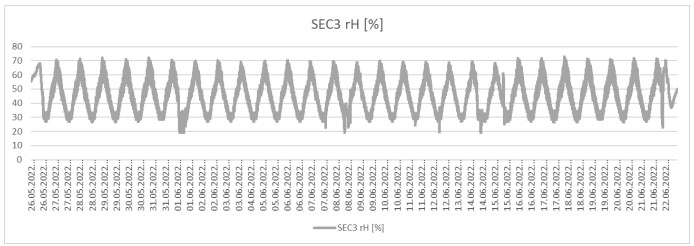

Supplement: S2 Fig — The following figures show the process of (a) temperature in [°C] and (b) relative humidity (rH) in [%] over the 4 weeks of testing. SEC1, SEC2 and SEC3 refers to the three 3 × 3 m2 chambers, each representing a different elevation (1,500, 2,500, and 4,000 m) and air pressure (85, 75, and 62 kPa). The graphs show the temperature and rH trends inside each chamber from the t0 (26.05) to the t2 (22.06). (DOCX) [file pone.0317590.s002.docx]
