# Supplementary material for: Short-term impact of low air pressure on plants’ functional traits
Source: PLoS One. 2025 Jan 15;20(1):e0317590. doi: 10.1371/journal.pone.0317590 (PMC11734969; doi:10.1371/journal.pone.0317590)
Supplement: S3 Fig — CO2 concentration in [ppm] inside the chambers (SEC1, blue line SEC2, orange line and SEC3, green line) from the t0 (26.05) to the t2 (22.06). (DOCX) [file pone.0317590.s003.docx]

**S3 Fig. CO2 variation in the chambers**. CO2 concentration in [ppm] inside the chambers (SEC1, *blue* line SEC2, *orange* line and SEC3, *green* line) from the t0 (26.05) to the t2 (22.06).

**
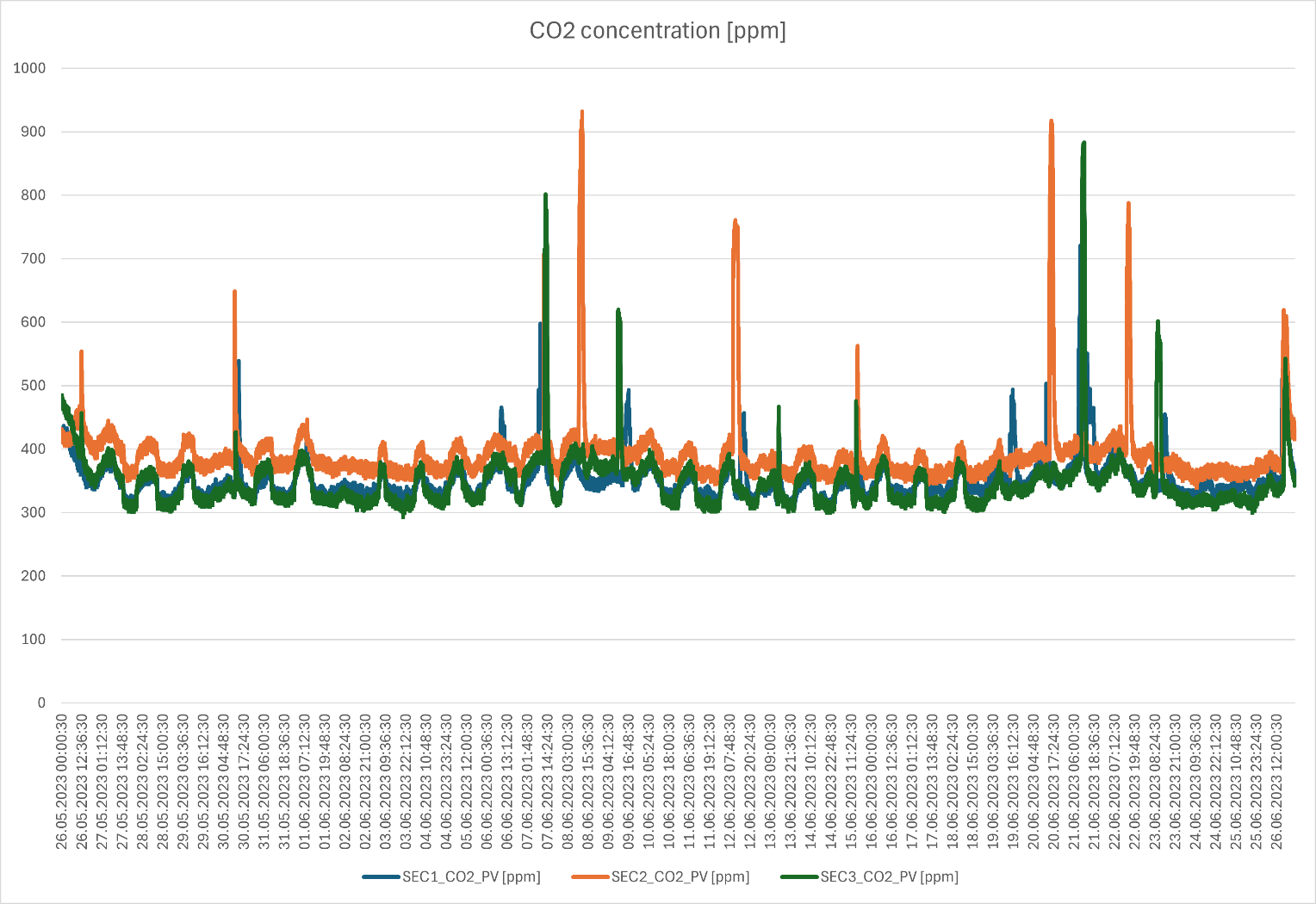
**
