# Supplementary material for: Short-term impact of low air pressure on plants’ functional traits
Source: PLoS One. 2025 Jan 15;20(1):e0317590. doi: 10.1371/journal.pone.0317590 (PMC11734969; doi:10.1371/journal.pone.0317590)
Supplement: S4 Fig — The scheme illustrates the design of each chamber. In each chamber, 60 pots were distributed across two benches. Each bench had five replicates per condition (five individuals × two water treatments × three species, n = 30). Light blue dots represent the wet treatment (0.1 L per pot each 48 h), and red dots represent the dry treatment (0.033 L per pot each 48 h). (DOCX) [file pone.0317590.s004.docx]

**S4 Fig. Schematic design representation.** The scheme illustrates the design of each chamber. In each chamber, 60 pots were distributed across two benches. Each bench had five replicates per condition (five individuals × two water treatments × three species, n = 30). Light blue dots represent the wet treatment (0.1 L per pot each 48 h), and red dots represent the dry treatment (0.033 L per pot each 48 h).

**
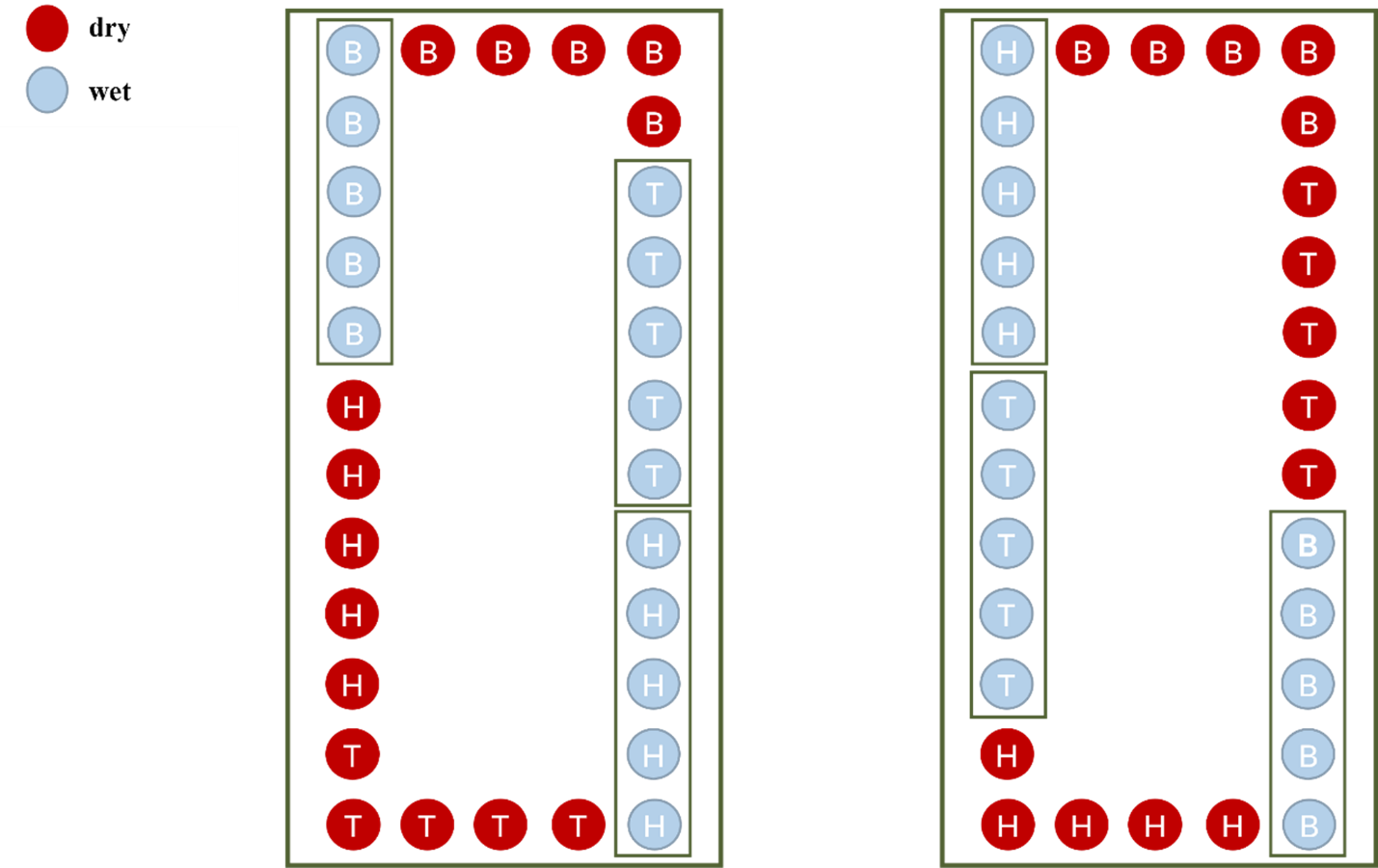
**

*Note: B: Brachypodium rupestre, H: Hieracium pilosella, T: Trifolium pratense.*
