# Supplementary material for: Short-term impact of low air pressure on plants’ functional traits
Source: PLoS One. 2025 Jan 15;20(1):e0317590. doi: 10.1371/journal.pone.0317590 (PMC11734969; doi:10.1371/journal.pone.0317590)
Supplement: S5 Fig — Effects of elevation on C content in aboveground biomass of Trifolium pratense (n = 20), Hieracium pilosella (n = 20), and Brachypodium rupestre (n = 20). The blue, yellow, and purple boxplot indicate the three air pressures tested in the chambers (85, 75, and 62 kPa). Lowercase letters indicate significant differences according to the post hoc test comparison (p < 0.05). Dots out of the whisker interval represent outliers. (DOCX) [file pone.0317590.s005.docx]

**S5 Fig. Effect of low air pressure on aboveground C content.** Effects of elevation on C content in aboveground biomass of *Trifolium pratense* (*n* = 20), *Hieracium pilosella* (*n* = 20), and *Brachypodium rupestre* (*n* = 20). The blue, yellow, and purple boxplot indicate the three air pressures tested in the chambers (85, 75, and 62 kPa). Lowercase letters indicate significant differences according to the post hoc test comparison (p < 0.05). Whiskers extend to the minimum and maximum values within 1.5 times the IQR from Q1 and Q3, respectively. Dots out of the whisker interval indicate outliers.

**
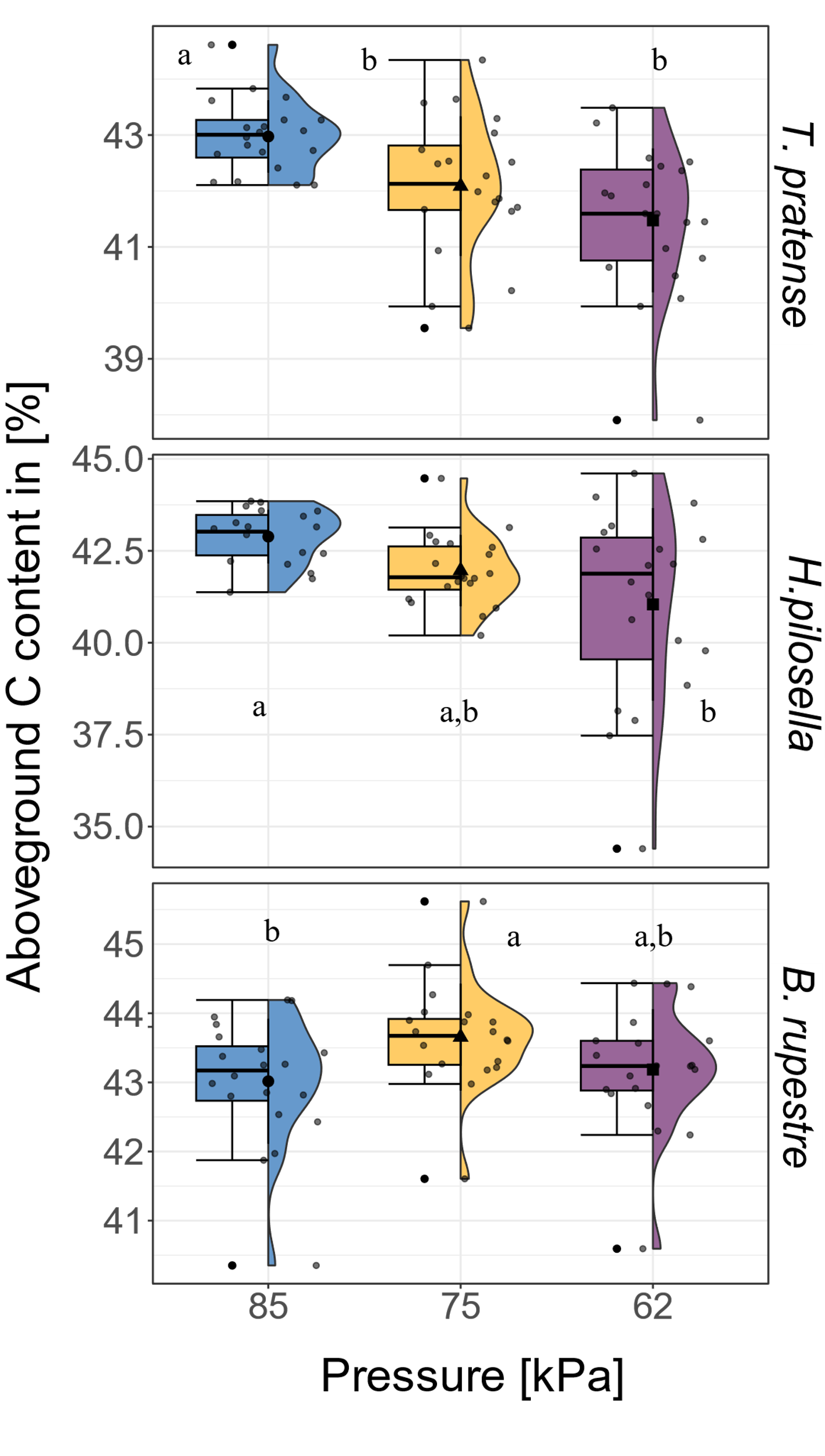
**
