# Supplementary material for: Short-term impact of low air pressure on plants’ functional traits
Source: PLoS One. 2025 Jan 15;20(1):e0317590. doi: 10.1371/journal.pone.0317590 (PMC11734969; doi:10.1371/journal.pone.0317590)
Supplement: S6 Fig — Interactive effects of pressure [kPa] and water treatment on (a) chlorophyll content (n = 20) and (b) N content in aboveground biomass (n = 10) of T. pratense and H. pilosella, at t2. The blue, yellow, and purple boxplot indicate the three different air pressures tested in the chambers (85, 75, and 62 kPa). Lowercase letters indicate significant differences according to the post hoc test comparison (p < 0.05). Dots out of the whisker interval represent outliers. (DOCX) [file pone.0317590.s006.docx]

**S6 Fig Combined low air pressure and water treatment effect on Chlorophyll and Nitrogen content in *T. pratense* and *H. pilosella*.** Interactive effects of pressure [kPa] and water treatment on ***(a)*** chlorophyll content (n = 20) and ***(b)*** N content in aboveground biomass (n = 10) of *T. pratense* and *H. pilosella,* at t2. The blue, yellow, and purple boxplot indicate the three different air pressures tested in the chambers (85, 75, and 62 kPa). Lowercase letters indicate significant differences according to the post hoc test comparison (p < 0.05). Whiskers extend to the minimum and maximum values within 1.5 times the IQR from Q1 and Q3, respectively. Dots out of the whisker interval indicate outliers.

**
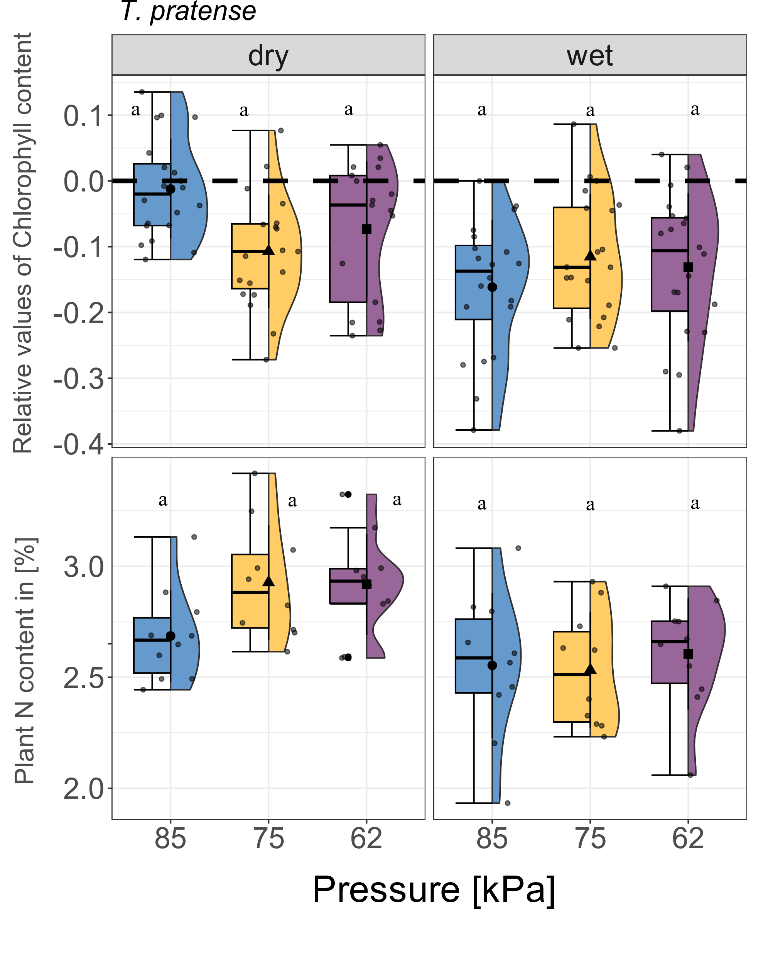
**

**
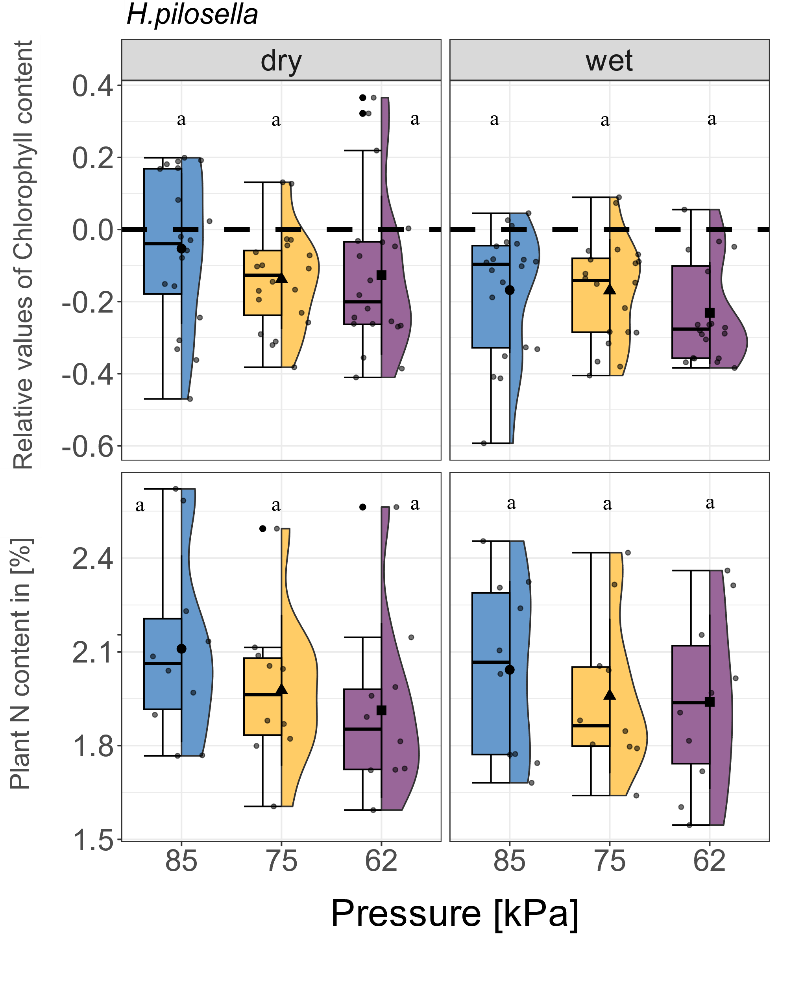
**
