# Supplementary material for: Short-term impact of low air pressure on plants’ functional traits
Source: PLoS One. 2025 Jan 15;20(1):e0317590. doi: 10.1371/journal.pone.0317590 (PMC11734969; doi:10.1371/journal.pone.0317590)
Supplement: S7 Fig — Effects of water treatment on SLA in (a) Trifolium pratense and (b) Brachypodium rupestre. (c) Mean values of specific leaf area (SLA). The interactive effect of water treatment (dry and wet) and time on SLA in (a) Trifolium pratense (n = 20) and (b) Brachypodium rupestre (n = 20). Relative values for t1 and t2 compared to t0 (black dotted line) are reported in the figures. Lowercase letters indicate significant differences according to the post hoc test comparison (p < 0.05). Dots out of the whisker interval represent outliers. (c) Mean values of SLA in [cm2 g-1] ± sd. (DOCX) [file pone.0317590.s007.docx]

**S7 Fig. Effects of water treatment on SLA in (a) *Trifolium pratense* and (b) *Brachypodium rupestre***. **(c)** **Mean values of specific leaf area (SLA).** The interactive effect of water treatment (dry and wet) and time on SLA in ***(a)*** *Trifolium pratense* (*n* = 20) and ***(b)*** *Brachypodium rupestre* (*n* = 20). Relative values for t1 and t2 compared to t0 (black dotted line) are reported in the figures. Lowercase letters indicate significant differences according to the post hoc test comparison (*p* < 0.05). Whiskers extend to the minimum and maximum values within 1.5 times the IQR from Q1 and Q3, respectively. Dots out of the whisker interval indicate outliers.(c) Mean values of SLA in [cm^2^ g^-1^] ± sd.

***
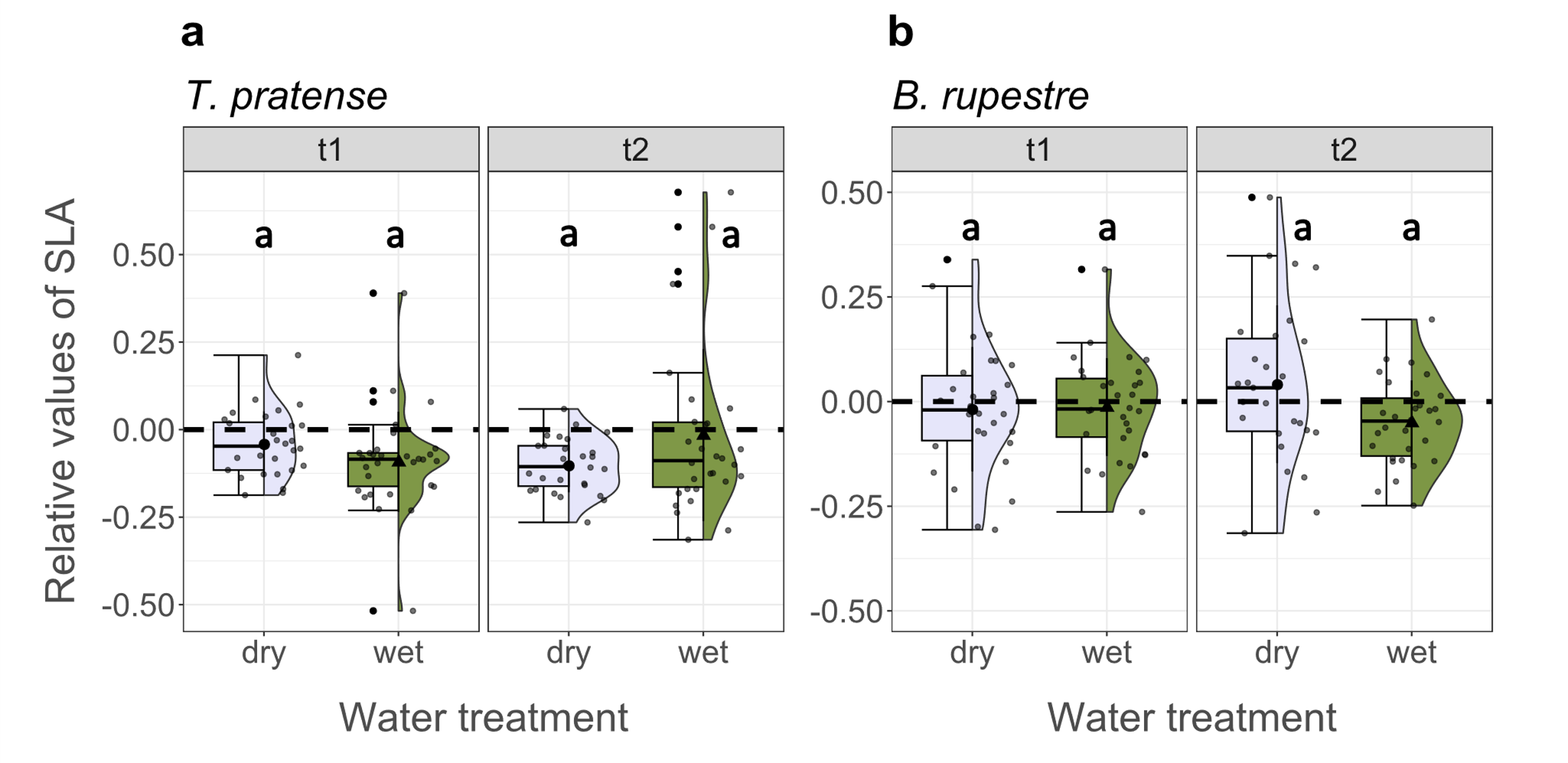
***

**c**

| Species | Water treatment | time | | |
| --- | --- | --- | --- | --- |
|  |  | **t_0_** | **t_1_** | **t_2_** |
| *Trifolium pratense* | dry | 257.200 ± 30.90 | 235.01 ± 41.37 | 209.51 ± 29.15 |
|  | wet | 270.48 ± 97.13 | 216.43 ± 36.49 | 211.46 ± 29.44 |
|  |  |  |  |  |
| *Brachypodium rupestre* | dry | 251.79 ± 60.23 | 240.75 ± 43.65 | 262.69 ± 82.27 |
|  | wet | 253.48 ± 39.47 | 247.33 ± 40.65 | 229.58 ± 40.01 |
