# Supplementary material for: Short-term impact of low air pressure on plants’ functional traits
Source: PLoS One. 2025 Jan 15;20(1):e0317590. doi: 10.1371/journal.pone.0317590 (PMC11734969; doi:10.1371/journal.pone.0317590)
Supplement: S1 Table — The SMALL CUBE (internal dimensions, 2.8 × 3 × 2.8 m (L × W × H)) consists of three smaller simulation chambers that can independently replicate the different environmental conditions present in the Alpine region. Three independent chambers were accessible via a common airlock. All environmental parameters were combined simultaneously to simulate complex scenarios. In this table, we show the achievable range for each parameter from minimum to maximum. The structure is also equipped with laboratories for sample preparation and analysis. (source: Structure—terraXcube (eurac.edu)). (DOCX) [file pone.0317590.s008.docx]

**S1 Table. Technical sheet, SMALL CUBE.** The SMALL CUBE *(internal dimensions, 2.8 × 3 × 2.8 m (L × W × H))* consists of three smaller simulation chambers that can independently replicate the different environmental conditions present in the Alpine region. Three independent chambers were accessible via a common airlock. All environmental parameters were combined simultaneously to simulate complex scenarios. In this table, we show the achievable range for each parameter from minimum to maximum. The structure is also equipped with laboratories for sample preparation and analysis. (*source:* [*Structure - terraXcube (eurac.edu)*](https://terraxcube.eurac.edu/structure/))

| Maximum simulated altitude | 4,000 m ± 10 m ( ~ 13,000 ft) |
| --- | --- |
| Temperature Range  According to IEC 60068-3-5 | -20…+50 °C ( ± 1°C in time ± 2 °C in space) |
| Temperature Rate of Change  According to IEC 60068-3-5 | ± 0,5 °C/min (cooling & heating) |
| Relative Humidity  T > 4°C and according to IEC 60068-3-6 | 10...100% ± 3% |
| Humidity rate of Change  T > 4°C and according to IEC 60068-3-6 | 0.4%/ min cooling; 0.8%/ min heating |
| Precipitation | Rain: 0–20 mm/h (Rainwater too) |
| Light | Full solar spectrum 280–900 nm, intensity 2,500 μmol/m^2^s |
| CO_2_ Control | 400–1,000 ppm |
