# Supplementary material for: Short-term impact of low air pressure on plants’ functional traits
Source: PLoS One. 2025 Jan 15;20(1):e0317590. doi: 10.1371/journal.pone.0317590 (PMC11734969; doi:10.1371/journal.pone.0317590)
Supplement: S2 Table — Daily cycle of temperature (T), relative humidity (Rh), vapor pressure deficit (VPD), light intensity (light), and photosynthetic photon flux density (PPFD) at hourly intervals, in three Ecotron chambers. PPFD values are measured by a light sensor directly in all three chambers. The elevation (air pressure) treatment was exchanged between the three chambers every 7 days clockwise. (DOCX) [file pone.0317590.s009.docx]

**S2 Table. Test report.** Daily cycle of temperature (T), relative humidity (Rh), vapor pressure deficit (VPD), light intensity (light), and photosynthetic photon flux density (PPFD) at hourly intervals, in three Ecotron chambers. PPFD values are measured by a light sensor directly in all three chambers. The elevation (air pressure) treatment was exchanged between the three chambers every 7 days clockwise.

| WHOLE time slot |  | |  | |  | |  |  |
| --- | --- | --- | --- | --- | --- | --- | --- | --- |
| Time slot | **T [/°C]** | **rH [/%]** | | **VPD [kPa]** | | **Light [/%]** | | **PPFD [µmol/m^2^s^-1^]** |
| 00:00 - 01:00 | 15 | 55 | | 0.42 | | 0 | | 0 |
| 01:00 - 02:00 | 14 | 60 | | 0.38 | | 0 | | 0 |
| 02:00 - 03:00 | 13 | 62 | | 0.35 | | 0 | | 0 |
| 03:00 - 04:00 | 12.5 | 60 | | 0.35 | | 0 | | 0 |
| 04:00 - 05:00 | 12 | 57 | | 0.34 | | 0 | | 0 |
| 05:00 - 06:00 | 13 | 55 | | 0.37 | | 0 | | 0 |
| 06:00 - 07:00 | 14 | 50 | | 0.40 | | 10 | | 188 |
| 07:00 - 08:00 | 15.5 | 46 | | 0.44 | | 30 | | 565 |
| 08:00 - 09:00 | 17 | 44 | | 0.48 | | 50 | | 942 |
| 09:00 - 10:00 | 18.5 | 41 | | 0.52 | | 63 | | 1186 |
| 10:00 - 11:00 | 20 | 38 | | 0.55 | | 75 | | 1412 |
| 11:00 - 12:00 | 21 | 35 | | 0.57 | | 90 | | 1695 |
| 12:00 - 13:00 | 22 | 32 | | 0.58 | | 100 | | 1883 |
| 13:00 - 14:00 | 23 | 30 | | 0.59 | | 90 | | 1659 |
| 14:00 - 15:00 | 23.5 | 30 | | 0.61 | | 80 | | 1506 |
| 15:00 - 16:00 | 24 | 28 | | 0.60 | | 70 | | 1318 |
| 16:00 - 17:00 | 23 | 30 | | 0.59 | | 60 | | 1130 |
| 17:00 - 18:00 | 22.5 | 30 | | 0.57 | | 50 | | 941 |
| 18:00 - 19:00 | 21 | 35 | | 0.57 | | 25 | | 470 |
| 19:00 - 20:00 | 19.5 | 40 | | 0.54 | | 0 | | 0 |
| 20:00 - 21:00 | 18.5 | 43 | | 0.52 | | 0 | | 0 |
| 21:00 - 22:00 | 17 | 45 | | 0.48 | | 0 | | 0 |
| 22:00 - 23:00 | 16 | 47 | | 0.45 | | 0 | | 0 |
| 23:00 - 00:00 | 15.5 | 50 | | 0.44 | | 0 | | 0 |

T = temperature, rH = Relative Humidity, VPD = vapor pressure deficit, PPFD = photosynthetic photon flux density
