# Supplementary material for: Short-term impact of low air pressure on plants’ functional traits
Source: PLoS One. 2025 Jan 15;20(1):e0317590. doi: 10.1371/journal.pone.0317590 (PMC11734969; doi:10.1371/journal.pone.0317590)
Supplement: S3 Table — Mean values in [mmol m-2 s-1] ± sd of stomatal conductance for Trifolium pratense (n = 20), Hieracium pilosella (n = 20) and Brachypodium rupestre (n = 20) at the beginning (t0), after two (t1) and four (t2) weeks since the start of the experiment at 85, 75, and 62 kPa. (DOCX) [file pone.0317590.s010.docx]

**S3 Table. Mean values of stomatal conductance.** Mean values in [mmol m^-2^ s^-1^] ± sd of stomatal conductance for *Trifolium pratense* (*n* = 20), *Hieracium pilosella* (*n* = 20) and *Brachypodium rupestre* (*n* = 20) at the beginning (*t_0_*), after two (*t_1_*) and four (*t_2_*) weeks since the start of the experiment at 85, 75, and 62 kPa.

| Species | Pressure [kPa] | time | | |
| --- | --- | --- | --- | --- |
|  |  | **t_0_** | **t_1_** | **t_2_** |
| *Trifolium pratense* | 85 | 174.06 ± 98.30 | 96.97 ± 33.34 | 102.01 ± 64.67 |
|  | 75 | 169.31 ± 102.53 | 160.35 ± 128.9 | 139.18 ± 61.88 |
|  | 62 | 110.96 ± 42.24 | 103.44 ± 62.02 | 172.14 ± 79.43 |
|  |  |  |  |  |
| *Hieracium pilosella* | 85 | 237.63 ± 118.45 | 207.52 ± 96.88 | 275.65 ± 185.45 |
|  | 75 | 255.95 ± 218.12 | 332.07 ± 265.3 | 467.85 ± 69.19 |
|  | 62 | 139.26 ± 63.94 | 181.02 ± 76.81 | 323.17 ± 11.70 |
|  |  |  |  |  |
| *Brachypodium rupestre* | 85 | 53.59 ± 34.05 | 79.31 ± 41.65 | 64.94 ± 59.62 |
|  | 75 | 57.75 ± 48.79 | 76.77 ± 42.79 | 61.33 ± 21.20 |
|  | 62 | 56.52 ± 20.45 | 77.95 ± 45.59 | 93.87 ± 61.52 |
