# Supplementary material for: Short-term impact of low air pressure on plants’ functional traits
Source: PLoS One. 2025 Jan 15;20(1):e0317590. doi: 10.1371/journal.pone.0317590 (PMC11734969; doi:10.1371/journal.pone.0317590)
Supplement: S4 Table — Mean values in [mg m2] ± sd of chlorophyll content for Trifolium pratense (n = 20), Hieracium pilosella (n = 20) and Brachypodium rupestre (n = 20) at the beginning (t0), after two (t1) and four (t2) weeks since the start of the experiment at 85, 75, and 62 kPa. (DOCX) [file pone.0317590.s011.docx]

**S4 Table. Mean values of chlorophyll content.** Mean values in [mg m^2^] ± sd of chlorophyll content for *Trifolium pratense* (*n* = 20)*, Hieracium pilosella* (*n* = 20) *and Brachypodium rupestre* (*n* = 20) at the beginning (*t_0_*), after two (*t_1_*) and four (*t_2_*) weeks since the start of the experiment at 85, 75, and 62 kPa.

| Species | Pressure [kPa] | time | | | |
| --- | --- | --- | --- | --- | --- |
|  |  | | **t_0_** | **t_1_** | **t_2_** |
| *Trifolium pratense* | 85 | | 481.85 ± 64.51 | 450.35 ± 39.26 | 364.70 ± 75.11 |
|  | 75 | | 500.15 ± 53.38 | 410.70 ± 58.02 | 390.79 ± 53.66 |
|  | 62 | | 485.60 ± 49.16 | 383.89 ± 76.12 | 407.47 ± 76.45 |
|  |  | |  |  |  |
| *Hieracium pilosella* | 85 | | 600.20 ± 109.50 | 572.40 ± 77.6 | 412.25 ± 154.72 |
|  | 75 | | 621.80 ± 101.19 | 548.50 ± 78.73 | 373.90 ± 69.19 |
|  | 62 | | 623.85 ± 112.62 | 399.85 ± 58.96 | 481.30 ± 68.39 |
|  |  | |  |  |  |
| *Brachypodium rupestre* | 85 | | 533.05 ± 49.88 | 388.75 ± 47.63 | 357.78 ± 97.69 |
|  | 75 | | 557.15 ± 73.98 | 381.40 ± 43.17 | 400.80 ± 65.73 |
|  | 62 | | 493.200 ± 65.35 | 398.70 ± 58.96 | 347.37 ± 68.39 |
