# Supplementary material for: Short-term impact of low air pressure on plants’ functional traits
Source: PLoS One. 2025 Jan 15;20(1):e0317590. doi: 10.1371/journal.pone.0317590 (PMC11734969; doi:10.1371/journal.pone.0317590)
Supplement: S5 Table — Mean values of specific leaf area (SLA) in [cm2 g-1] ± sd for Trifolium pratense, Hieracium pilosella and Brachypodium rupestre at the beginning (t0), after two (t1) and four (t2) weeks since the start of the experiment at 85, 75, and 62 kPa. Measurements refer to five leaves per sample in T. pratense (n = 20), one leaf per sample in H. pilosella (n = 20) and two leaves per sample in B. rupestre (n = 20). (DOCX) [file pone.0317590.s012.docx]

**S5 Table. Mean values of specific leaf area (SLA).** Mean values of specific leaf area (SLA) in [cm^2^ g^-1^] ± sd for *Trifolium pratense*, *Hieracium pilosella* and *Brachypodium rupestre* at the beginning (*t_0_*), after two (*t_1_*) and four (*t_2_*) weeks since the start of the experiment at 85, 75, and 62 kPa. Measurements refer to five leaves per sample in *T. pratense* (*n* = 20), one leaf per sample in H. pilosella (*n* = 20) and two leaves per sample in *B. rupestre* (*n* = 20).

| Species | Pressure  [kPa] | time | | | |
| --- | --- | --- | --- | --- | --- |
|  |  | **t_0_** | **t_1_** | | **t_2_** |
| *Trifolium pratense* | 85 | 244.36 ± 39.44 | 206.74 ± 39.09 | | 202.32 ± 30.59 |
|  | 75 | 263.54 ± 42.76 | 231.22 ± 42.29 | | 211.16 ± 23.15 |
|  | 62 | 283.77 ± 108.59 | 239.67 ± 30.41 | | 218.51 ± 31.75 |
|  |  |  | |  |  |
| *Hieracium pilosella* | 85 | 160.12 ± 23.96 | 124.31 ± 16.13 | | 140.69 ± 24.86 |
|  | 75 | 156.60 ± 13.31 | 133.52 ± 17.99 | | 140.08 ± 22.34 |
|  | 62 | 137.38 ± 15.25 | 131.37 ± 24.15 | | 135.74 ± 30.92 |
|  |  |  | |  |  |
| *Brachypodium rupestre* | 85 | 246.27 ± 46.38 | 246.98 ± 32.17 | | 233.04 ± 27.19 |
|  | 75 | 257.30 ± 63.52 | 240.07 ± 48.59 | | 243.78 ± 53.95 |
|  | 62 | 254.33 ± 40.79 | 245.06 ± 45.19 | | 258.41 ± 95.49 |
