# Supplementary material for: Short-term impact of low air pressure on plants’ functional traits
Source: PLoS One. 2025 Jan 15;20(1):e0317590. doi: 10.1371/journal.pone.0317590 (PMC11734969; doi:10.1371/journal.pone.0317590)
Supplement: S6 Table — Mean values in [mg m2] ± sd of chlorophyll content for Brachypodium rupestre (n = 20) at the beginning (t0) and after four weeks (t2) since the start of the experiment at 85, 75, and 62 kPa in dry (d) and wet (w) treatments. (DOCX) [file pone.0317590.s013.docx]

**S6 Table. Mean values of chlorophyll content for different water treatment.** Mean values in [mg m^2^] ± sd of chlorophyll content for *Brachypodium rupestre* (*n* = 20) at the beginning (*t_0_*) and after four weeks (*t_2_*) since the start of the experiment at 85, 75, and 62 kPa in dry (d) and wet (w) treatments.

| Time | Pressure [kPa] | | Water treatment | | | |
| --- | --- | --- | --- | --- | --- | --- |
|  | | **d** | | **w** | |  |
|  | 85 | | 534.30 ± 58.09 | | 531.80 ± 43.29 | |
| *t_0_* | 75 | | 565.40 ± 73.85 | | 548.90 ± 77.14 | |
|  | 62 | | 488.00 ± 56.47 | | 498.40 ± 75.95 | |
|  | 85 | | 428.75 ± 75.57 | | 301.00 ± 74.33 | |
| *t_2_* | 75 | | 446.80 ± 56.33 | | 354.80 ± 35.28 | |
|  | 62 | | 335.56 ± 68.22 | | 358.00 ± 70.37 | |
